# RNA-Seq Profiling of a Defective Seed Coat Mutation in *Glycine max* Reveals Differential Expression of Proline-Rich and Other Cell Wall Protein Transcripts

**DOI:** 10.1371/journal.pone.0096342

**Published:** 2014-05-14

**Authors:** Anupreet Kour, Anne M. Boone, Lila O. Vodkin

**Affiliations:** Department of Crop Sciences, University of Illinois, Urbana, Illinois, United States of America; Agriculture and Agri-Food Canada, Canada

## Abstract

The plant cell wall performs a number of essential functions including providing shape to many different cell types and serving as a defense against potential pathogens. The net pattern mutation creates breaks in the seed coat of soybean (*Glycine max*) because of ruptured cell walls. Using RNA-Seq, we examined the seed coat transcriptome from three stages of immature seed development in two pairs of isolines with normal or defective seed coat phenotypes due to the net pattern. The genome-wide comparative study of the transcript profiles of these isolines revealed 364 differentially expressed genes in common between the two varieties that were further divided into different broad functional categories. Genes related to cell wall processes accounted for 19% of the differentially expressed genes in the middle developmental stage of 100–200 mg seed weight. Within this class, the cell wall proline-rich and glycine-rich protein genes were highly differentially expressed in both genetic backgrounds. Other genes that showed significant expression changes in each of the isoline pairs at the 100–200 mg seed weight stage were xylem serine proteinase, fasciclin-related genes, auxin and stress response related genes, TRANSPARENT TESTA 1 (TT1) and other transcription factors. The mutant appears to shift the timing of either the increase or decrease in the levels of some of the transcripts. The analysis of these data sets reveals the physiological changes that the seed coat undergoes during the formation of the breaks in the cell wall.

## Introduction

Plant cells are surrounded by cell walls which provide tensile strength, mechanical support, protection from insects and pathogens, prevention of water loss, and that participate in cell to cell communication [1], [2], [3], [4]. The cell walls differ in composition in different plant parts but the basic composition of the cell wall includes cellulose, hemicellulose, pectins, and small proportions of structural proteins including proline-rich and glycine-rich proteins. The seed coat is the outer protective layer of a seed and develops from the integument originally surrounding the ovule and is maternal in origin. The seed coat provides protection of embryo and endosperm from mechanical injuries, insects, bacteria, and fungi, and desiccation of the seed. Seed coats of different species vary in structure and composition including extensive differentiation of cell layers into specialized cell types. These cells may accumulate large quantities of substances including mucilage or pigments depending on plant species.

The seed coat, or testa, of the mature soybean (*Glycine max*) has been well characterized, and contains features in common with the majority of the legumes: an epidermal layer of palisade cells, or macrosclereids, a sub-epidermal layer of hourglass cells, or osteosclereids, a few layers of parenchyma, and an aleurone layer [5], [6], [7]. Soybean seeds begin to form on the plant at the R4 stage when the parent plant has between 13 and 20 leaf nodes [8]. At this stage, the seed is going through multiple cycles of cell division as well as tissue differentiation. Invertase in the seed coat cleaves sucrose giving rise to hexose which surrounds the embryo of the minuscule soybean seed [9]. The seeds grow at a rapid rate between the R4 and R7 stages as they accumulate carbon, nitrogen, and seed storage proteins. Seeds increase in size from 25 mg to 500 mg fresh weight during the R5 and R6 stages. Towards the end of the R6 stage, nutrient accumulation in the seed begins to decrease. By R7 the seed has amassed almost all the dry weight it will acquire and contains approximately 60% moisture and has begun to yellow. After reaching the maximum fresh weight, near 400–500 mg, the seed readies itself for desiccation and begins to lose total fresh weight. By the R8 stage, most of the pods and seeds have browned and are dry [8].

Intact seed coats are preferred but a mutation known as the “net pattern” in soybean results in defective seed coats that have ruptured by maturity to expose the cotyledons underneath. The trait has been backcrossed to create isolines in two different genetic backgrounds; however, very little is known about the histology or molecular nature of the trait. Using RNA blots, we have previously reported a developmental delay in the decline of transcripts for a specific proline-rich protein (PRP1) of the cell wall leading to higher levels of this transcript in the defective seed coats at the middle weight range of 100–200 mg seed weight [10].

The effect on PRP1 transcript levels was assumed to be a downstream effect of the net pattern mutation. In this report, we present the global gene expression analysis of the net pattern cell wall mutant in soybean using transcriptome analyses of three different stages of seed development at 50–100 mg, 100–200 mg, and 400–500 mg. By comparing RNA-Seq data from the two lines containing the defective seed coat trait compared to their standard counterpart isolines using Bowtie alignments [11] to the soybean genes models from the sequenced soybean genome [12], 364 significantly differentially expressed genes in common between the two isolines were revealed, many of which were involved in cell wall processes. These data aid in determining perturbations in the physiological development of the plant cell wall which affects agronomically important traits and is a source of bioenergy [13].

## Results

### Phenotype Comparisons of Isoline Pairs with Standard and Defective Seed Coats

**Figure 1:**
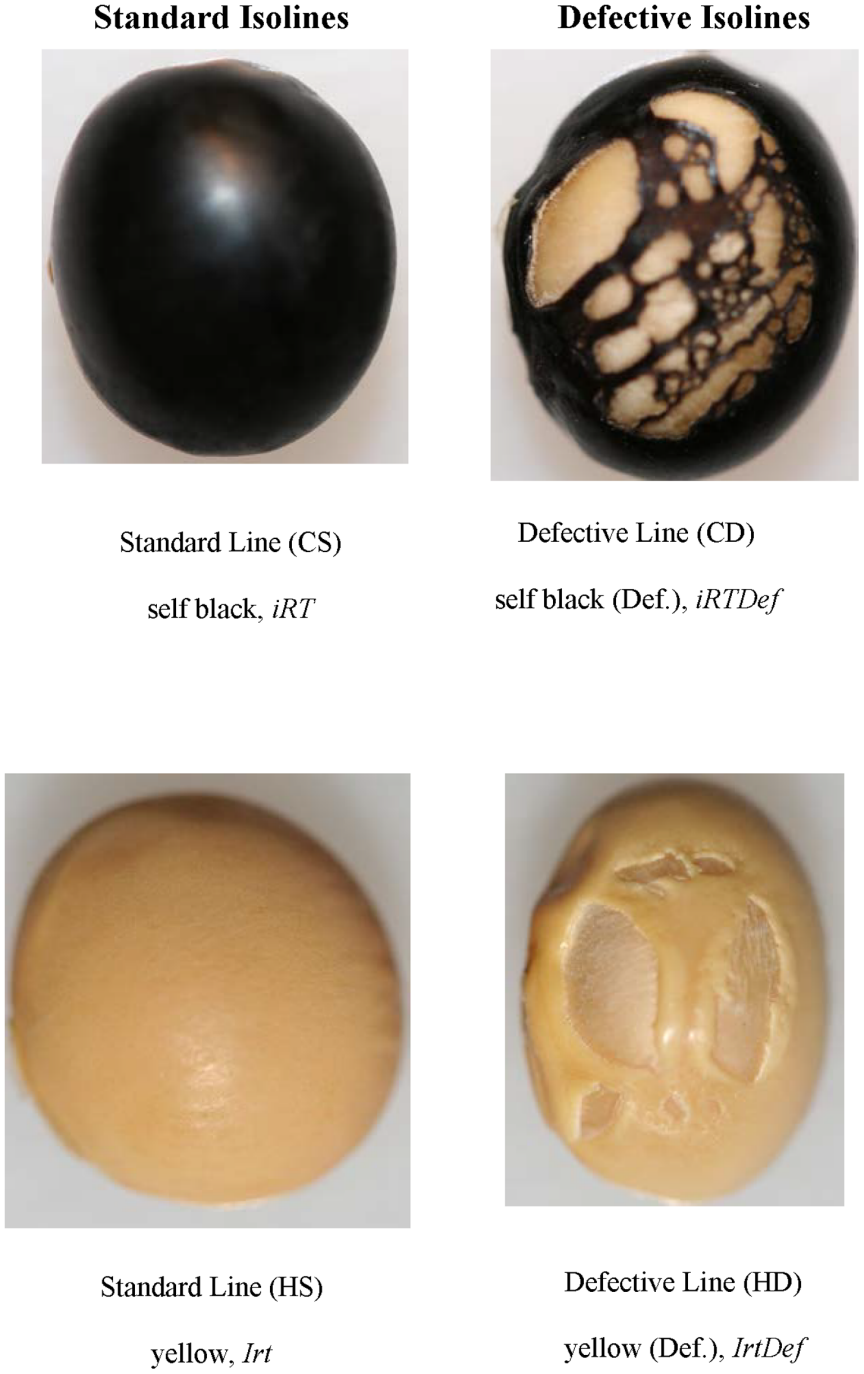
Comparisons of the Defective Seed Coat Mutation in Soybean. The genotypes and phenotypes of the isolines used in both Clark and Harosoy backgrounds are shown. Representative seed comparing Clark Standard (CS) vs. Clark Defective (CD) and Harosoy Standard (HS) vs. Harosoy Defective (HD) illustrate the defective seed coat mutation exhibiting cracks on seed coat in either a pigmented or non-pigmented genetic background. Refer to Table 1 for additional information.

The isolines in the background of cultivar Clark are referred to as Clark standard (CS) which exhibits an intact seed coat and Clark defective (CD) that contains the defective seed coat mutation that had been backcrossed into the Clark standard line. Likewise, Harosoy standard (HS) is compared to the Harosoy defective (HD) isoline that contains the defective seed coat mutation. The genotypes and phenotypes of these two isoline pairs is presented in Figure 1. As shown in Figure 1, all the layers of seed coat are ruptured in the mutant isolines. Genetically, mutant isolines contain an unknown gene or set of genes here designated as "Def" for defective seed coat. This abbreviation is not meant to convey an official locus designation. This mutation was transferred during the 1960's by R. L. Bernard of the USDA/ARS by selection of the trait during repetitive backcrossing into both a Clark (with a black seed background) and a Harosoy (yellow seed background). The defective seed coat phenotype is more striking on the black background and is sometimes referred to as a “net” pattern [10]. The phenotypes of the two lines along with their alleles controlling seed color [14], [15], [16], [17] are shown in Figure 1 and Table 1. The two lines are available from the USDA germplasm collection, Urbana, Illinois, as shown in Table 1.

**Table 1:** Summary of 12 RNA-Seq Libraries Constructed from Seed Coats of Clark and Harosoy Standard and Defective Seed Coat Isolines at Three Stages of Immature Seed Development.

Isolines	Genotype	Phenotype	USDA #	Internal Lab #	Seed wt. (mg)	Raw reads (M)	Sequencing platform	Read length (bp)
CS	*i R T*	Self-black	PI 547474	UC9	50–100	52.1	HiSeq2000	100
CS	*i R T*	Self-black	PI 547474	UC9	100–200	62.3	GAII	75
CS	*i R T*	Self-black	PI 547474	UC9	400–500	50.5	HiSeq2000	100
CD	*i R T* Def	Defective black	Clark 5143	UC412	50–100	68.5	HiSeq2000	100
CD	*i R T* Def	Defective black	Clark 5143	UC412	100–200	41.1	GAII	75
CD	*i R T* Def	Defective black	Clark 5143	UC412	400–500	31.2	HiSeq2000	100
HS	*I r t*	Yellow	PI 548573	UC501	50–100	46.4	HiSeq2000	100
HS	*I r t*	Yellow	PI 548573	UC501	100–200	38.7	GAII	75
HS	*I r t*	Yellow	PI 548573	UC501	400–500	49.2	HiSeq2000	100
HD	*I r t* Def	Defective yellow	L67–2246	UC508	50–100	49.4	HiSeq2000	100
HD	*I r t* Def	Defective yellow	L67-2246	UC508	100–200	38.2	GAII	75
HD	*I r t* Def	Defective yellow	L67-2246	UC508	400–500	37.7	HiSeq2000	100

The USDA line number and PI accession numbers by which the isolines are searchable in the USDA GRIN (Germplasm Resources Information Network) database are shown. The molecular nature is known for the *I* alleles [14], [15] that control distribution of pigment and the *R* [16], and *T* loci [17] that affect seed color phenotype. RNA was extracted from seed coats dissected from immature seed of the indicated fresh weight ranges. The total number of 75- or 100-nt raw reads from each RNA-Seq library is shown. The pictures of each phenotype of the defective seed coat lines are shown in Figure 1. The raw and processed data for the 12 sequencing samples is available as accession series GSE54903 at the National Center for Biotechnology Information. (Abbreviations: CS: Clark Standard, CD: Clark Defective, HS: Harosoy Standard, HD: Harosoy Defective).

### Transcriptome Comparisons of Clark and Harosoy Standard Seed Coats to Defective Seed Coats from the Mutant Isolines

Next-generation high throughput sequencing of the transcriptome (RNA-Seq) was used and the cDNA libraries were constructed from seed coats from 50–100 mg, 100–200 mg and 400–500 mg seed weight stages in standard and mutant isolines of Clark and Harosoy cultivars. Each library was sequenced on the Illumina GAII or HiSeq 2000 platforms. The number of raw sequence reads obtained from all four isolines, at different seed weight stages are presented in Table 1. The 75-bp or 100-bp reads were aligned to 78,773 target Glyma models from the soybean reference genome [12] using Bowtie [11]. The RNA-Seq data were normalized in reads per kilobase of gene model per million mapped reads (RPKM) considering gene length as parameter [18].

As presented in Table S1, 18% and 50% of the genes were expressed in the seed coat of Clark and Harosoy isolines at the cutoff points of above 10 RPKM and 1 RPKM, respectively. For inclusion in further analyses, all genes that showed ≥5 RPKM in either standard or defective isoline as well a ≥2 fold differential expression between standard and defective isolines were included in further functional annotation analyses. An overview of the number of these genes is presented in Table 2 which shows that a total of approximately 1,300 out of 78,773 genes in the soybean genome showed differential expression in the seed coats of either the Clark or Harosoy isolines in at least one stage of development. The p-values for differential expression were obtained by analysis with the DESeq package on hit count data from comparisons between Clark and Harosoy standard and defective isolines. The gene models that showed p-values ≤0.05 were considered as significantly differentially expressed. These differentially expressed genes along with gene model numbers, annotations, RPKM values, basemean, and p-values are presented in Tables S2–S4. The graphical presentation of expression levels of these differentially expressed genes in standard and defective isolines are presented in Figures S1 & S2. The majority of the differentially expressed seed coat genes were expressed at less than 50 RPKM in both Clark and Harosoy backgrounds.

**Table 2:** Total Number of Differentially Expressed Genes in the Seed Coats of the Clark and Harosoy Isoline Pairs at Different Seed Weight Stages.

Genotypes	Number of differentially expressed genes			
	50–100 mg	100–200 mg	400–500 mg	Total
Clark	720	173	417	1310
Harosoy	48	156	1068	1272
Both	57	265	42	364
Opposite	9	4	96	109

The number of differentially expressed genes in Clark or Harosoy standard versus the defective seed coat isoline that meet the criteria of ≥2 fold differential expression, ≥5 RPKM and p-value ≤0.05 by DESeq analysis. Both: the number of differentially expressed genes shared in common between both the Clark and Harosoy isoline pairs and also the expression levels of these genes are in the same direction *i.e.* if expression of a particular gene was higher in Clark wild-type and lower in the defective isoline, it was also higher in Harosoy wild type isoline and lower in Harosoy defective isoline. Opposite: If a particular gene showed higher expression in the standard isoline of Clark compared to the Clark defective, but it showed higher expression in the defective Harosoy compared to the standard Harosoy line.

### Functional Classification of Genes Differentially Expressed in Seed Coats of both Clark and Harosoy Isolines

**Figure 2:**
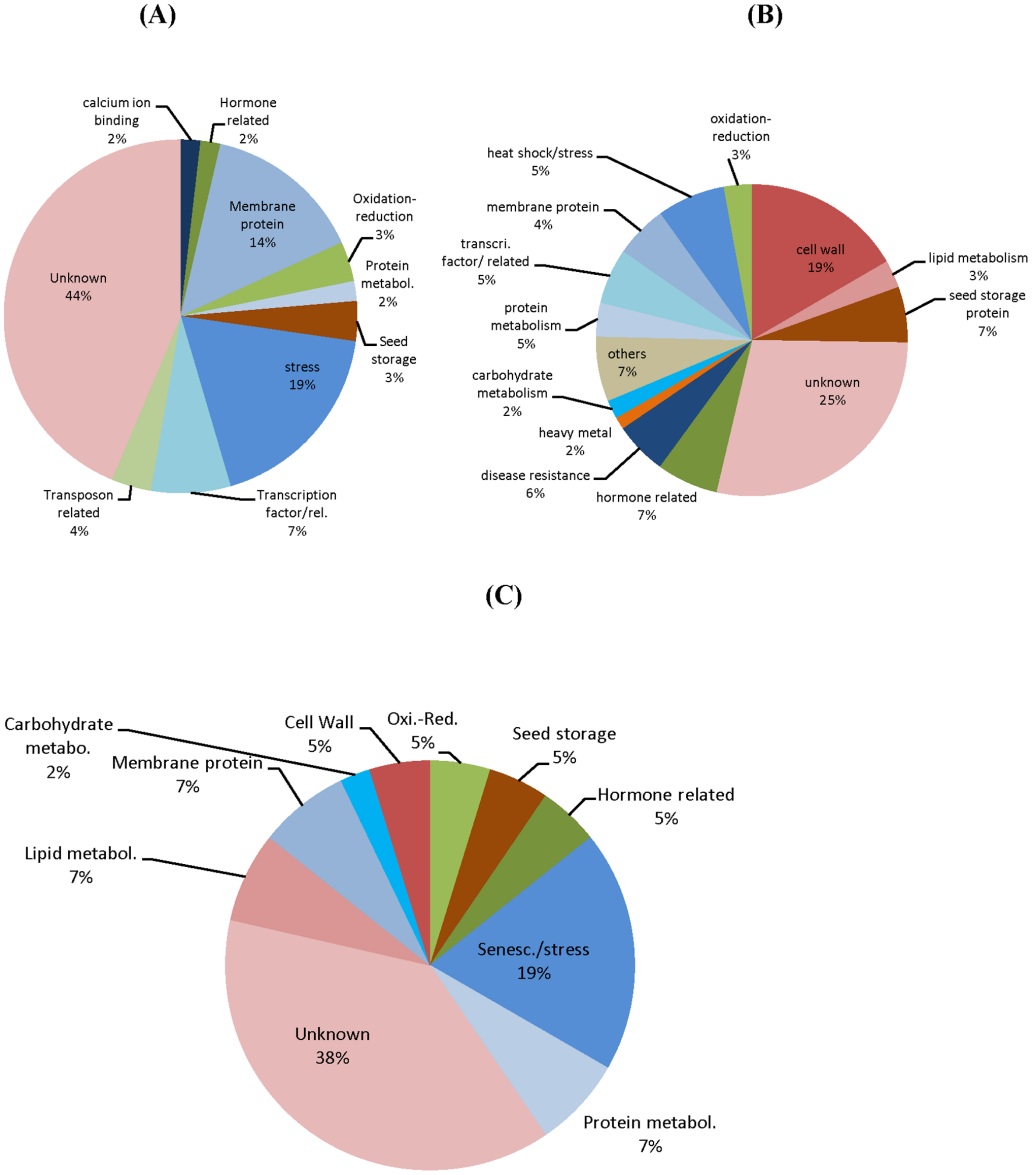
The Distribution of Genes Differentially Expressed in Standard and Defective Seed Coats of Both Clark and Harosoy Backgrounds During Three Stages of Immature Seed Coat Development. The genes that were differentially expressed in both Clark and Harosoy backgrounds at seed weight ranges of either (A) 50–100 mg (B) 100–200 mg and (C) 400–500 mg. Differential expression is defined as genes with ≥5RPKM expression, ≥2 fold differential expression, and p value ≤0.05. Differential expression of cell wall related genes comprise 19% of the total genes at the 100–200 mg seed weight stage and 5% of the 400–500 mg stage.

**Figure 3:**
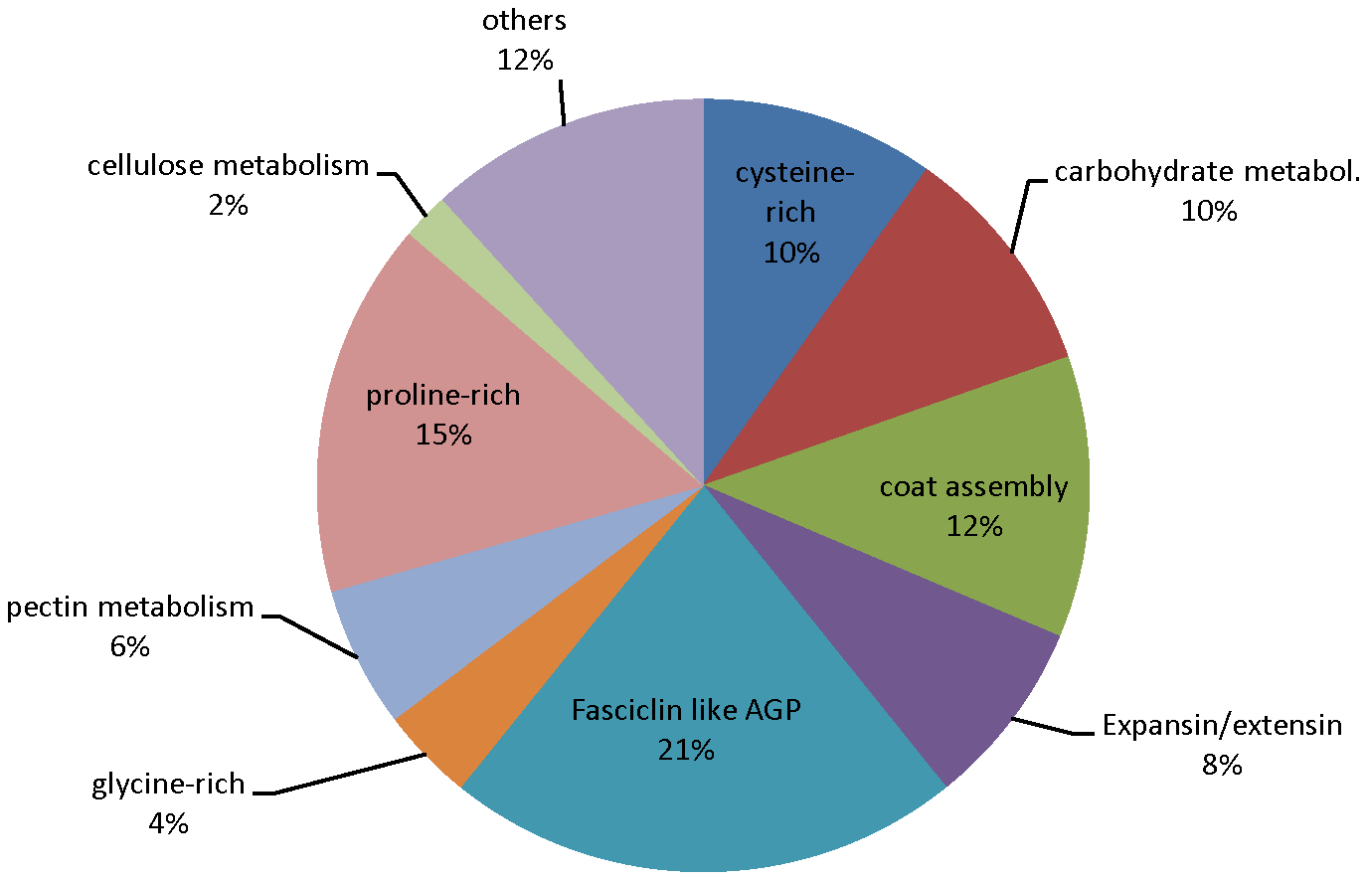
The Distribution of Cell wall Related Genes Differentially Expressed Between Standard and Defective Seed Coats in Both Clark and Harosoy Backgrounds at the 100–200 mg Seed Weight Stage. The distribution of 51 differentially expressed genes into different cell wall structural component categories is shown with genes having annotations related to fasciclin (21%) and protein rich proteins (15%) predominating.

As shown in Table 2, a total of 364 genes were differentially expressed in at least one of the three different seed weight stages in both Clark and Harosoy isolines. The detailed information on these genes is presented in Table S3 and distribution of these genes into different broad functional categories is presented in Figure 2. Out of these 364 genes, there were 265 that showed significantly different expression in the 100–200 mg weight range while only 57 and 42 genes varied in the 50–100 and 400–500 mg weight ranges, respectively. A representative selection of 29 genes is presented in Tables 3 and 4. In addition, 248 of these 265 varied only at the 100–200 mg stage and did not show differential expression at the 50–100 or 400–500 mg weight range. Thus, the maximum number of significantly differentially expressed genes in common in both the Clark and Harosoy backgrounds are in the 100–200 mg seed weight range. In the 50–100 mg weight range, there were no differentially expressed genes with cell wall annotations (Figure 2A), while at the 100–200 mg and 400–500 mg weight range there were 19% and 5% of the differentially expressed genes related to possible cell wall functions, respectively (Figures 2B and 2C). The 51 cell wall related genes at the 100–200 mg stage were further divided into 10 different categories based on cell wall structural components (Figure 3). The maximum number (21%) of genes fall under the fasciclin-like arabinogalactan proteins that have been reported to affect tensile strength of the cell wall [19]. Proline-rich proteins are the second most abundant category at 15%, while cysteine-rich proteins, expansin/extensin-related proteins, and glycine-rich proteins (GRPs) represent 10%, 8%, and 4% of the differentially expressed cell wall proteins in this developmental stage (Figure 3).

**Table 3:** Selected Differentially Expressed Genes in the Clark Isoline Pair at the 100–200 mg Seed Weight Stage Using RPKM and DESeq Analysis of RNA-Seq Data.

a) Overexpressed in Defective		RNA-Seq (RPKM)			DESeq (Base Mean)		
Model	Annotation	CS	CD	CD/CS	CS	CD	p value
Glyma13g27050.1	unknown	4.90	68.73	14.02	567	7298	2.77E-13
**Glyma09g12200.1** *	**proline rich protein (PRP1)**	874	6181	7.07	94605	613411	1.22E-08
Glyma13g01720.1	transparent testa 1	1.59	9.84	6.10	130	737	6.46E-06
Glyma14g35140.1	transparent testa 1	1.60	9.55	5.90	112	614	1.66E-05
Glyma08g46790.1	plant self-incompatibility protein S1	17.10	82.41	4.82	655	2893	1.03E-05
Glyma11g27320.1	unknown	43.47	205	4.72	1809	7836	7.33E-06
Glyma05g25180.1	senescence-associated protein 29	8.00	36.24	4.50	579	2403	2.61E-05
Glyma08g08200.1	senescence-associated protein 29	10.60	45.29	4.20	784	3070	4.27E-05
Glyma09g41940.1	cytochrome P450 monooxygenase	3.11	12.48	4.01	635	2333	0.0001
Glyma17g09110.1	strictosidine synthase protein	95.78	338	3.53	10219	33034	0.0002
Glyma05g07630.1	strictosidine synthase protein	105	312	2.97	11557	31638	0.001
Glyma04g02660.1	GAST1 protein homolog 1	421	1222	2.90	22553	60062	0.002
Glyma04g02460.2	Xylem serine proteinase 1	189	434	2.29	42068	88490	0.017
**b) Overexpressed in Standard**							
**Model**	**Annotation**	**CS**	**CD**	**CS/CD**	**CS**	**CD**	**p value**
Glyma12g34160.1	glycinin subunit G7	40.19	1.46	27.6	2844	94.4	1.79E-18
Glyma10g35840.1	Unknown	523	32.38	16.16	36331	2060	2.48E-16
Glyma10g35870.1	ADR12 protein	729	45.18	16.13	38268	2174	2.52E-16
Glyma04g38640.1	amino acid permease AAP3	25.69	1.6	16.09	4805	274	2.52E-15
Glyma10g39170.1	lipid transfer family protein	21.29	2.32	9.17	3995	399	4.81E-11
Glyma06g15410.1	calmodulin-binding protein	181	22.16	8.16	15411	1730	8.87E-11
Glyma07g28610.1	glycine rich protein	27.31	3.85	7.08	1617	209	1.75E-08
Glyma10g03400.1	sucrose-binding protein	9.58	1.75	5.48	436	72.89	2.88E-05
Glyma02g42630.1	glycine rich protein	30.91	5.7	5.42	1521	257	6.63E-07
Glyma15g23830.1	proline rich protein	144	26.61	5.41	10955	1857	7.80E-08
Glyma09g12250.1	proline rich protein	608	115	5.31	82019	14167	7.25E-08
**Glyma09g12260.1** *	**proline rich protein (PRP2)**	809	174	4.66	120651	23748	5.13E-07
Glyma09g24410.1	Hsp90 protein	23.31	5.43	4.29	5297	1132	3.07E-06
Glyma05g21680.1	auxin-responsive GH3 product	8.63	2.05	4.19	1629	356	1.34E-05
Glyma13g36400.1	seed storage protein	239	67.03	3.57	19730	5061	2.32E-05
Glyma05g33200.1	defensin-like protein	192.12	54.13	3.54	10413	2689	2.83E-05

(a) Overexpressed in Clark Defective (CD) or (b) Overexpressed in Clark Standard (CS).

*Indicates Proline Rich Protein (PRP) genes that have had been reported to have major structural functions in the cell wall.

**Table 4:** Selected Differentially Expressed Genes in the Harosoy Isoline Pair at the 100–200 mg Seed Weight Stage Using RPKM and DESeq Analysis of RNA-Seq Data.

a) Overexpressed in defective		RNA-Seq (RPKM)			DESeq (Base Mean)		
Model	Annotation	HS	HD	HD/HS	HS	HD	p value
Glyma11g27320.1	unknown	0.54	80	80.00	16	2248	3.04E-14
Glyma17g09110.1	strictosidine synthase protein	2.33	177	75.96	174	12748	1.86E-13
Glyma08g46790.1	Plant self-incompatibility protein S1	0.26	17	65.38	7	438	3.53E-08
Glyma05g07630.1	strictosidine synthase protein	2.45	154	62.85	189	11508	1.09E-12
Glyma05g25180.1	senescence-associated protein 29	0.64	48	48	32	2363	3.11E-12
Glyma13g27050.1	unknown	0.01	47	47	0.98	3655	6.87E-22
Glyma04g02660.1	GAST1 protein homolog 1	16	600	37.50	608	21746	9.57E-11
**Glyma09g12200.1** *	**proline rich protein (PRP1)**	162	5890	36.35	12282	430912	8.71E-11
Glyma08g08200.1	senescence-associated protein 29	1.81	64	35.35	94	3192	6.58E-10
Glyma09g41940.1	cytochrome P450 monooxygenase	1.80	47	26.11	257	6537	3.35E-09
Glyma14g35140.1	transparent testa 1	0.78	15	15	38	699	2.23E-06
Glyma13g01720.1	transparent testa 1	0.75	13	13	43	719	3.53E-06
Glyma04g02460.2	Xylem serine protease	73	556	7.61	11434	83564	5.84E-05
**b)Overexpressed in Standard**							
**Model**	**Annotation**	**HS**	**HD**	**HS/HD**	**HS**	**HD**	**p value**
Glyma06g15410.1	calmodulin-binding protein	570	0.52	570	33949	30	2.32E-24
Glyma02g42630.1	glycine rich protein	530	0.19	530	18225	6	2.21E-26
Glyma07g28610.1	glycine rich protein	416	0.05	416	17234	2.05	3.68E-28
Glyma09g12250.1	proline rich protein	2369	6	395	223255	551	8.49E-21
**Glyma09g12260.1** *	**proline rich protein (PRP2)**	3438	9	382	358154	874	7.21E-21
Glyma15g23830.1	proline rich protein	423	1.45	292	22525	75	3.67E-19
Glyma10g39170.1	lipid transfer family protein	87	2.83	31	11353	359	3.67E-10
Glyma05g33200.1	defensin-like protein	621	26	24	23517	935	2.19E-09
Glyma09g24410.1	Hsp90 protein	36	2.54	14	5751	389	3.44E-07
Glyma04g38640.1	amino acid permease 3	34	2.51	14	4438	318	6.35E-07
Glyma12g34160.1	glycinin subunit G7	158	12	13	7812	576	5.94E-07
Glyma05g21680.1	auxin-responsive GH3 product	10	0.27	10	1361	35	1.88E-09
Glyma13g36400.1	seed storage protein	401	42	10	23097	2382	6.99E-06
Glyma10g03400.1	sucrose-binding protein	78	15	5	2467	454	0.0008
Glyma10g35840.1	Unknown	45.51	11.63	3.91	2208	545	0.005
Glyma10g35870.1	ADR12 protein	63.83	16.65	3.83	2342	590	0.006

(a) Overexpressed in Harosoy Defective (HD) or (b) Overexpressed in Harosoy Standard (HS).

*Indicates Proline Rich Protein (PRP) genes that have had been reported to have major structural functions in the cell wall.

A selection of 29 of the 364 differentially expressed genes at the 100–200 mg seed weight stage are shown in Table 3 (in Clark background) and Table 4 (in Harosoy background) along with their functional annotations, fold-changes in differential expression, and p-values. The data for all 364 genes are shown in Table S3. Six genes in Tables 3 and 4 have cell wall structural protein annotations, including proline-rich proteins PRP1 and PRP2 and glycine-rich proteins.

### Cell Wall Related Genes Expressed in Both Clark and Harosoy Seed Coats

**Figure 4:**
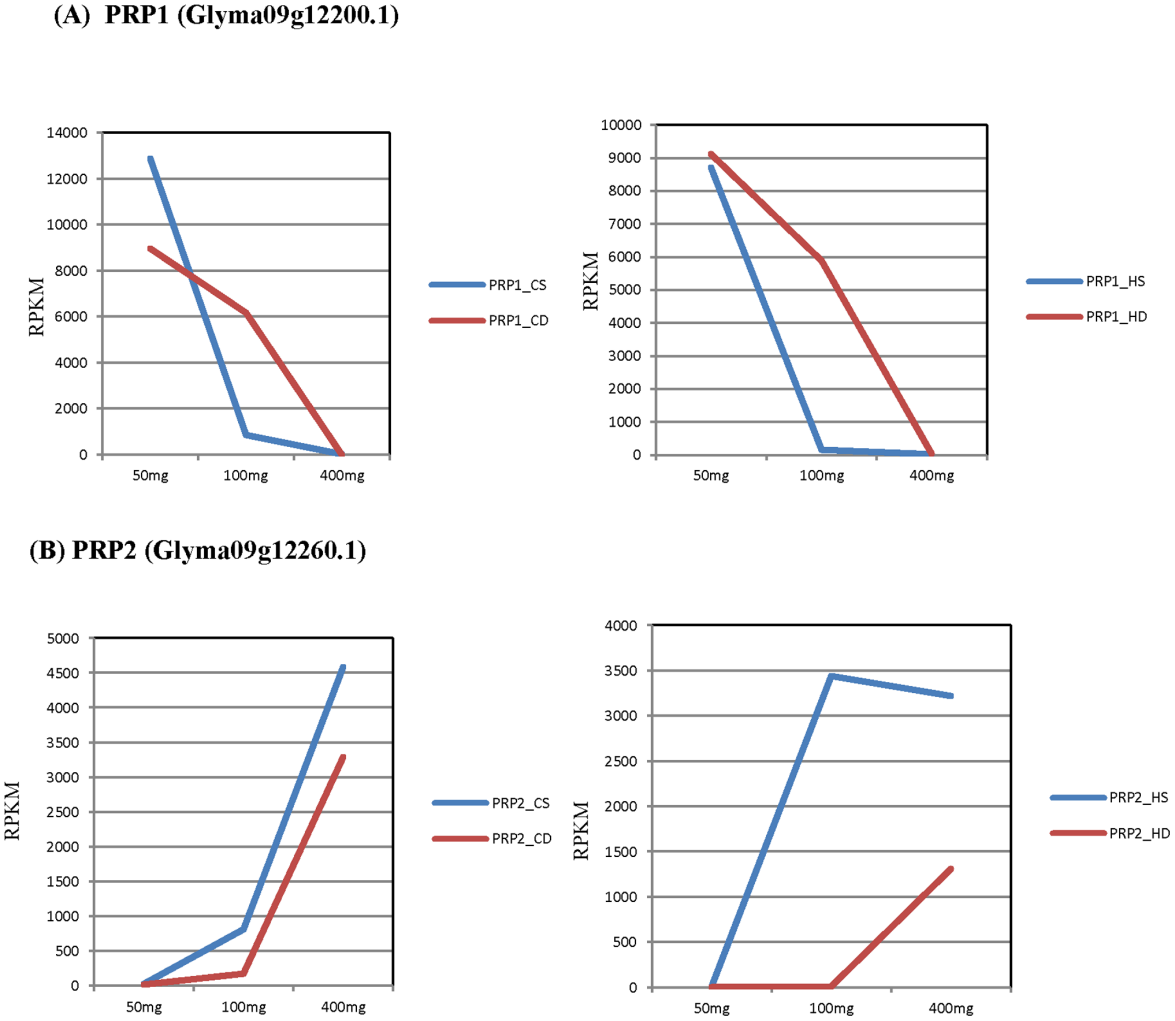
The Expression Levels of (A) PRP1 and (B) PRP2 Genes in Standard and Defective Seed Coats of either Clark or Harosoy Backgrounds at Three Stages of Seed Development. The blue line in the graphs represents expression level (RPKM) of these genes in seed coats of standard isolines and the red line represents expression levels in the defective seed coats at the 50–100 mg, 100–200 mg, and 400–500 mg seed weight ranges. Maximal differential expression of the genes between the standard and defective isolines was observed at the 100–200 mg seed weight stage. (Abbreviations: CS: Clark standard, CD: Clark defective, HS: Harosoy standard, HD: Harosoy defective).

As presented in Tables 3 & 4, PRP1 and PRP2 were two of the highly differentially expressed proline-rich protein genes in both Clark and Harosoy isolines. In the Harosoy isolines, both the RPKM levels and fold differences for these genes were higher as compared to Clark isolines. The expression of the PRP1 was higher earlier in development and declined as shown in Figure 4A. In contrast, PRP2 was expressed only at the later stages of seed development as seen in Figure 4B. The differential expression between the standard and defective seed coats was maximal for both PRP1 and PRP2 at the 100–200 mg weight range in Clark and Harosoy isolines. PRP1 transcripts appeared to decline in abundance faster in the standard seed coats of both Clark and Harosoy isolines leading to higher levels in the defective seed coats whereas PRP2 transcripts were always less abundant in the defective seed coats.

The expression levels of PRP1 and PRP2 at different seed weight stages were previously demonstrated by [10] using RNA blots in the Clark isoline. Our previous RNA blots agree with our present RNA-Seq data showing that PRP1 was expressed more abundantly in early development, whereas PRP2 was present in the older seed coats. These two Glyma models were called as “low confidence” gene models with no annotation by the Glyma gene model version 1.0 at the Phytozome database and as extensin-related genes in Glyma model version 1.1, whereas the non-redundant database of the National Center for Biotechnology Information (NCBI) annotated them as proline-rich proteins. We conducted manual annotations in the following manner to confirm them as the classically defined soybean PRP1 and PRP2 genes which are in NCBI. The nucleotide and amino acid sequences of PRP1 from NCBI (J02746) showed 100% match with Glyma09g12200.1 from the soybean genome (Figure S3), and the nucleotide sequence of SbPRP2 gene from NCBI (J05208) showed 98.8% similarity with Glyma09g12260.1 (Figure S4). There was a difference of 10 amino acids between the amino acid sequence of PRP2 in cultivars Wayne (amino acid sequence from NCBI) and Williams (amino acid sequence from the soybean genome). Both PRP1 and PRP2 genes are linked and separation between these genes is approximately 146 kb (Figure S5).

The graphical representations of the expression levels of the other differentially expressed genes shown in Tables 3 and 4 at all three different stages of seed development is presented in Figures S6 and S7, respectively, showing that all have the highest differential expression at the mid- developmental stages of 100–200 mg seed weight. Included is the pattern for several other proline and glycine-rich proteins and that of the xylem serine proteinase that is overexpressed in the defective seed coats (Figure S6-M). In many of these genes, those that are highly expressed in the 50–100 mg seed coats and then decline show a pattern similar to PRP1 in that they are overexpressed in the defective seed coats. Likewise, those that are overexpressed in the standard line are similar to PRP2 in that they generally have higher RPKM expression levels later in development.

Figure 3 shows that 21% of the cell wall related genes were annotated as fasciclin related. The graphical view of expression of these fasciclin-like arabinogalactan genes in the seed coats of both standard and defective seed coat isolines at three different stages of seed development is presented in Figure S8. All of the genes showed expression patterns with significant differential expression at 100–200mg seed weight stage and their transcript levels were higher in the defective as compared to the standard seed coats, as was the case with PRP1. Thus, all 11 of these genes were overexpressed in the defective seed coat. Figure S9 graphs the developmental profiles of the remaining 25 cell wall genes overexpressed in the defective isolines and Figure S10 shows the remaining 3 cell wall genes that were overexpressed in the standard seed coats.

It is clear from the 68 graphs of the gene expression patterns shown in Figures S6–S10 that the 100–200 mg weight range shows the highest differential expression and that many of the genes can be classified as either declining in expression or increasing in expression during seed coat development as is the case for the PRP1 and PRP2 patterns. In many cases, the net pattern mutation appears to increase expression of many of the cell wall related genes in the 100–200 mg weight range as for PRP1. In contrast, many of the genes that are overexpressed in the standard line appear to be increasing during late seed development in both standard and defective lines as does PRP2.

### Hormone Regulated Genes which were Differentially Expressed in Both Clark and Harosoy Backgrounds

**Figure 5:**
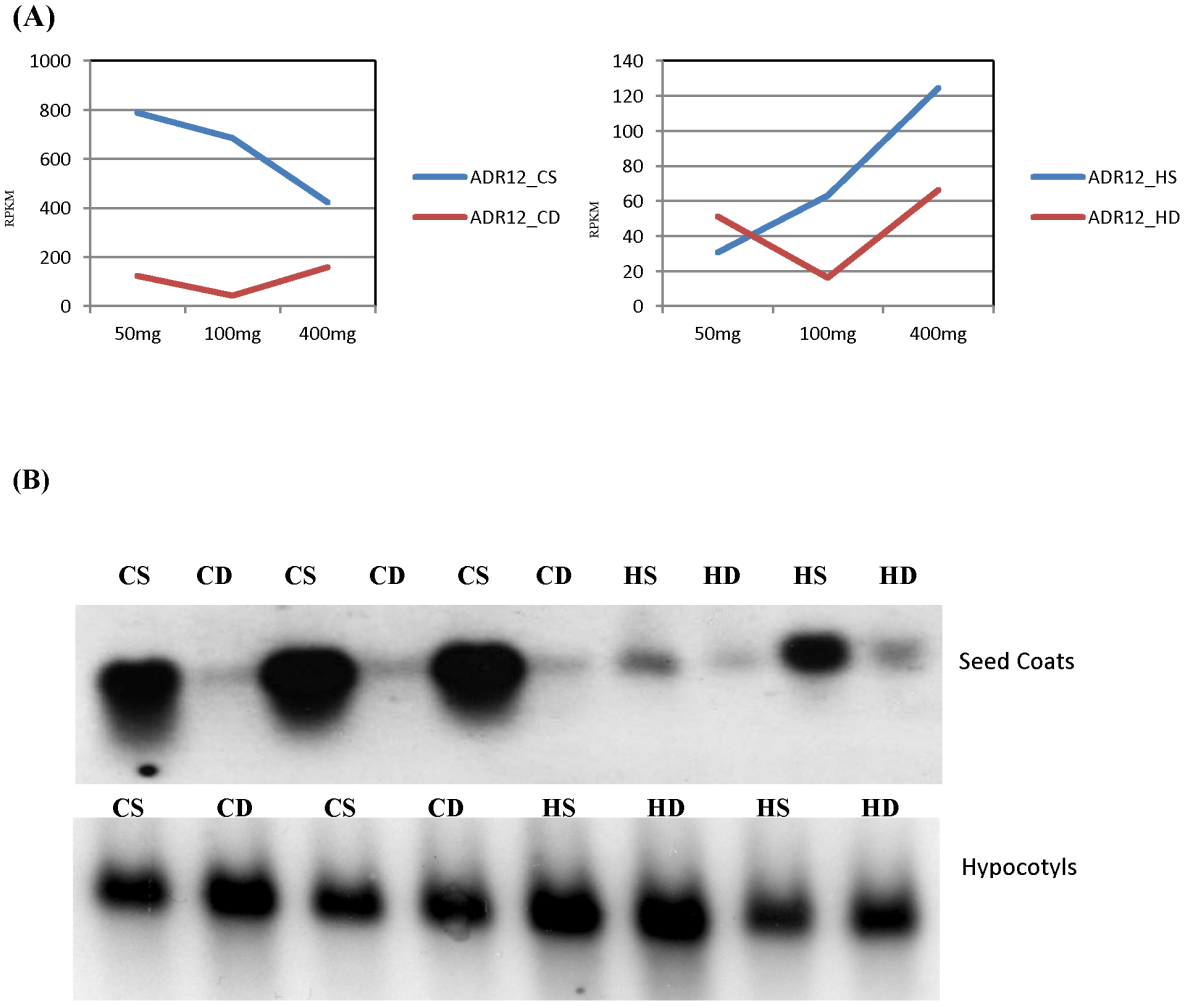
The Expression Levels of ADR12 (Glyma10g35870.1) Transcripts in Standard and Defective Seed Coat Isolines as Determined by RNA-Seq and RNA blots. (A) The levels of ADR12 (ADR12-2) transcripts (as RPKM) in seed coats of Clark (left) or Harosoy (right) isolines. The blue line in the graphs represents the expression level (RPKM) of this gene in seed coats of standard isolines and the red line represents expression levels in the defective seed coats at the 50–100 mg, 100–200 mg, and 400–500 mg seed weight ranges. (B, Top) The Northern blots of RNA from 100–200 mg soybean seed coats representing three independent extractions of the Clark and two independent samples of the Harsoy lines. (B, Bottom) The Northern blot of RNA from 4–5 days old soybean hypocotyls extracted from two independent samples of each line. Each gel lane was loaded with 10 ug of total RNA, electrophoresed, stained with ethidium bromide to confirm equal loadings, and blotted. The RNA blots were probed with Gm-r1088-6964, a EST clone representing ADR12 and corresponding to Glyma10g35870.1. Whereas there is differential expression in the seed coats of standard and defective lines, there was no difference in expression of ADR12 in the hypocotyls. (Abbreviations: CS: Clark Standard, CD: Clark defective, HS: Harosoy standard, HD: Harosoy defective).

There were 21 hormone regulated genes that were differentially expressed in the seed coat of wild type and defective isolines of both Clark and Harosoy background (Table S3) including representative auxin and gibberellin regulated genes that are shown in Tables 3 & 4. One gene model Glyma10g35870.1 showed the best match to the authentic ADR12 (auxin down regulated) gene from NCBI (S58482). It showed approximately 16-fold lower expression in the Clark defective seed coats and 4-fold lower expression expression in Harosoy defective seed coats (Tables 3 & 4). Auxin is an important phytohormone that plays several important roles in plant growth and development. The expression level of this gene at different stages of seed development in both standard and defective seed coat isolines is presented in Figure 5A for RNA-Seq data. In the Clark standard isoline, this gene showed higher expression in earlier stages, and there was continuous decrease in the expression level at later stages of seed development. There was significant differential expression at all the seed developmental stages (Figure 5A, left). In the Harosoy standard isoline, expression of ADR12 increased continuously with developmental stages and also increased in the defective seed coats at the two older stages, although the levels were significantly lower in the defective seed coats (Figure 5A, right). The expression pattern of ADR12 using RNA blots (Figure 5B, top) coincides well with the RNA-Seq data in that the defective seed coats have reduced expression especially in the Clark isolines. In addition, the blots confirm that the ADR12 is more highly expressed in standard seed coats of Clark than in Harosoy. As presented in the blots in Figure 5B, bottom panel, there was no differential expression observed from 4–5 days old soybean hypocotyls in standard and defective isolines of Clark and Harosoy. Thus, the differential expression of this gene is likely confined to the seed coats that manifest the defective cell wall structure.

The expression pattern of two other hormone regulated genes in Tables 3 and 4 that showed significant differential expression in the seed coat of wild type and defective isolines of both Clark and Harosoy backgrounds is presented in Figures S6-L and S7-N. The gene that showed a gibberellin regulated annotation had an expression pattern similar to PRP1 with higher expression in defective isolines as compared to the standard seed coats at 100–200 mg seed weight (Figure S6-L), while the other gene that had an auxin related annotation showed a different expression pattern with under expression in the defective isoline at the 100–200 mg seed weight stage (Figure S7-N).

### Genes Differentially Expressed between Standard and Defective Isolines in Either the Clark or Harosoy Backgrounds

Along with the genes that showed significant differential expression in the seed coats of both genetic backgrounds, there were genes that showed significant differential expression in either the Clark or Harosoy isolines. As presented in Table 2 and Table S2, there were approximately 1300 genes that showed differential expression in either the Clark or Harosoy background. These genes were divided into different broad functional categories based on functional annotations and presented in Figures S11, S12 and S13. The cell wall category is highest at the 100–200 mg stages and comprises 14% in Clark and 44% of the total differentially expressed genes in Harosoy seed coats. Another major category was transcription factor and transcription related genes (up to 11%). There were 109 genes with functional annotations that showed opposite differential expression (*ie*., if expression of a particular gene was higher in the standard isoline of Clark, then it was lower in the standard isoline of Harosoy) (Table S4).

### Transcription Factor Genes that were Differentially Expressed in Standard and Defective Seed Coat Isolines

**Figure 6:**
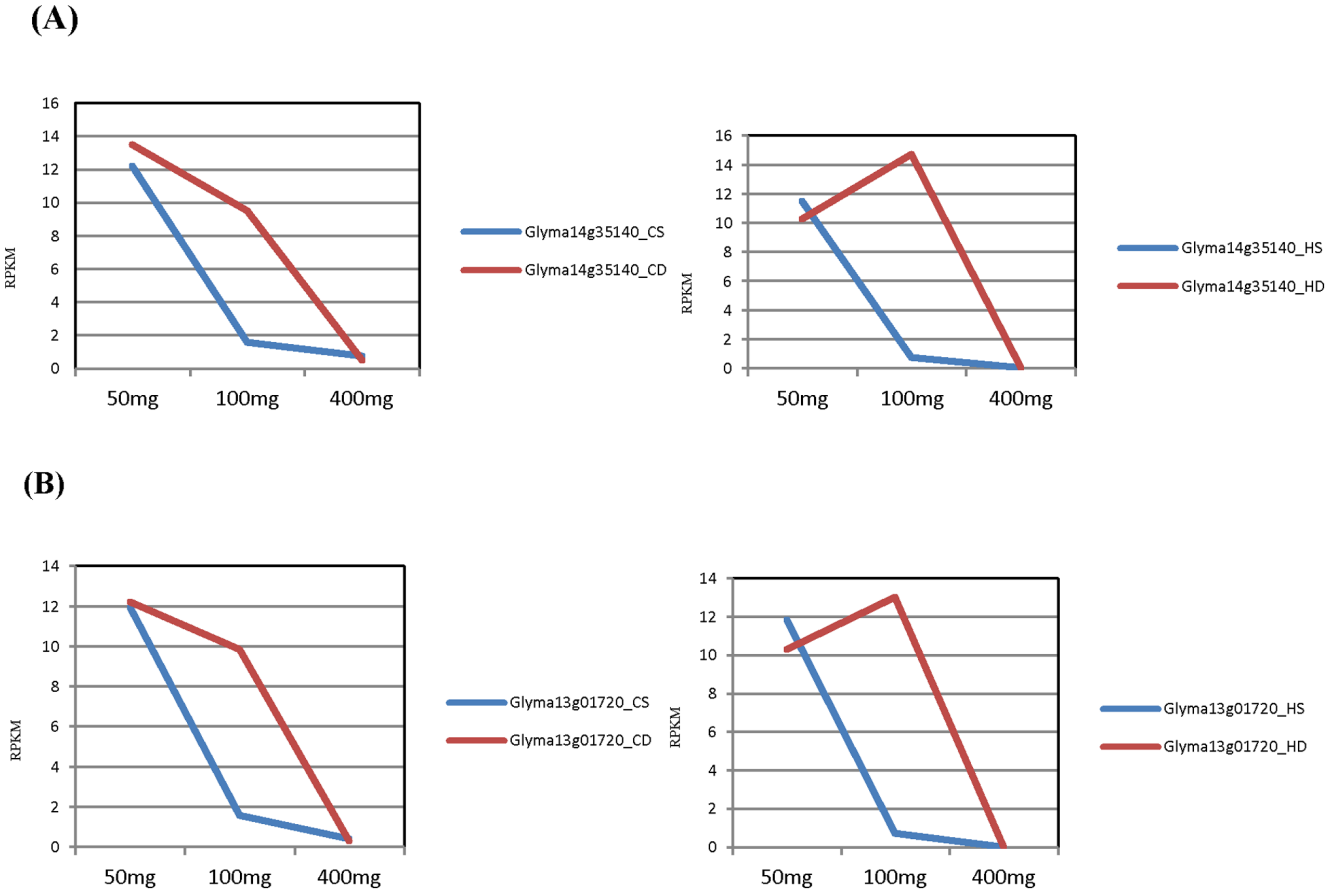
The Expression Levels of TRANSPARENT TESTA 1 (TT1) Genes in Standard or Defective Seed Coats of Clark and Harosoy Backgrounds. Both Glyma14g35140 (Figure A) and Glyma13g01720 (Figure B) gene models had TT1 annotations. The blue line in the graphs represents expression level (RPKM) of these genes in seed coats of standard isolines and the red line represents expression levels in the defective seed coats at the 50–100 mg, 100–200 mg, and 400–500 mg seed weight ranges. There was significant differential expression in these genes at 100–200mg seed weight range. (Abbreviations: CS: Clark standard, CD: Clark defective, HS: Harosoy standard, HD: Harosoy defective).

Two zinc finger domain (C2H2 type) containing protein genes that had TT1 (TRANSPARENT TESTA1) annotations were approximately 6-fold and 15-fold more highly expressed in the defective seed coats of the Clark and Harosoy lines, respectively, at the 100–200 mg seed weight stage (Tables 3 & 4). The expression level of these transcription factor genes at different stages of development is presented in Figure 6. These genes showed higher expression at the younger stage in both standard and defective isolines in the Clark background and then decreased with progressive seed weight stage. They increased somewhat in the Harosoy line at the 100–200 mg seed stage before declining.

**Figure 7:**
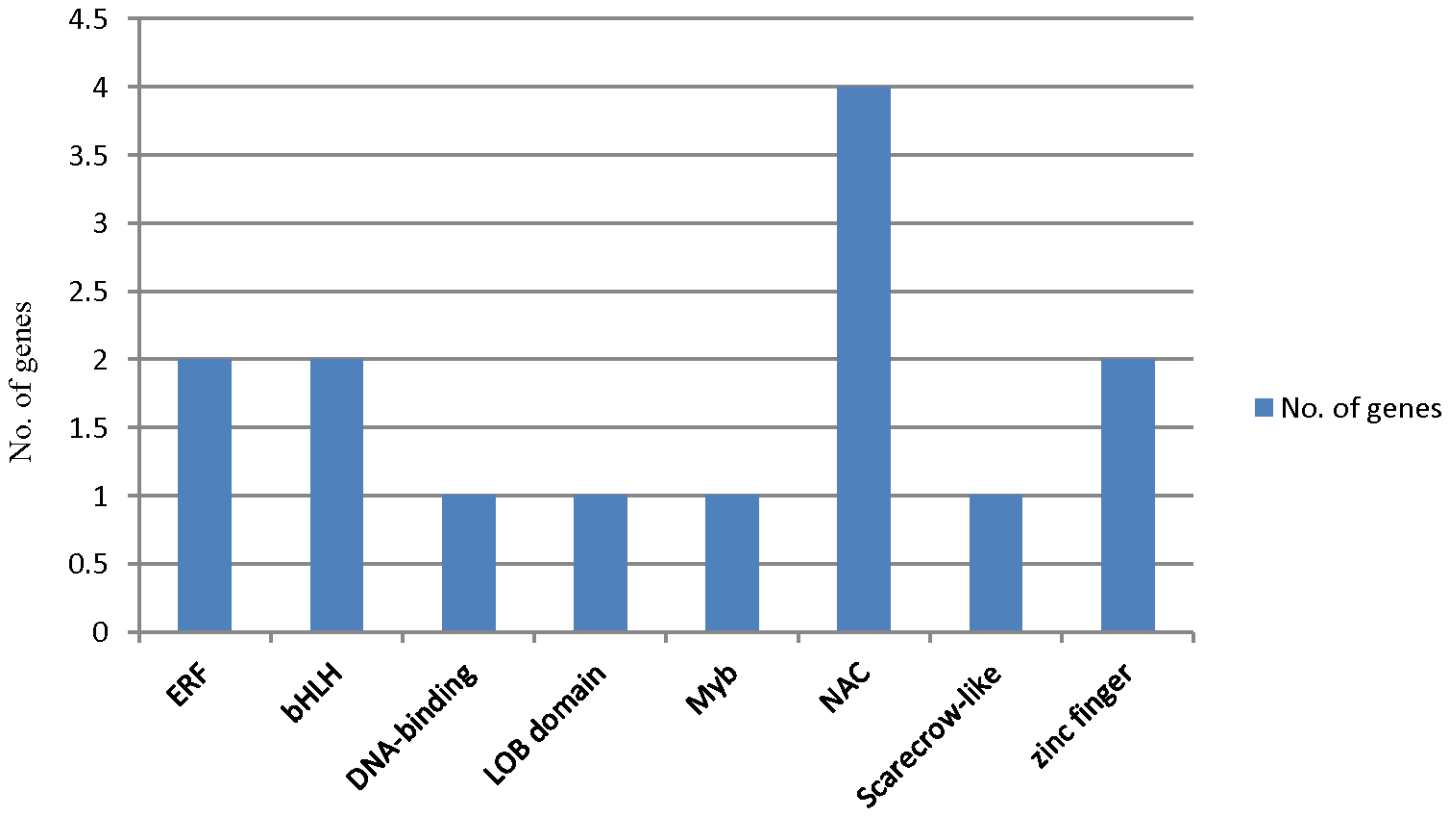
The Annotations of Differentially Expressed Transcription Factor Genes in Standard and Defective Seed Coats in Common Between Clark and Harosoy Backgrounds at the 100–200mg Seed Weight stage. The classification is shown of 14 significantly differentially expressed transcription factor genes (≥5RPKM, ≥2 fold differential expression and p-value ≤0.05) at the 100–200 mg seed weight stage that are in common between both the Clark and Harosoy backgrounds.

Along with the two TT1 gene models, there were 14 total transcription factor genes that showed differential expression in seed coats of standard and defective isolines in both of the Clark and Harosoy backgrounds at the 100–200 mg stage (Figure 7, Table S3). The most prominent category had NAC-related annotations. The other important classes of transcription factor genes were bHLH, zinc finger and ethylene responsive factor genes. The expression of these transcription factor genes at three stages of seed development is presented in Figure S14 and S15. There were two transcription factor genes (Myb and bHLH) that showed expression patterns similar to TT1 and PRP1 (Figure S14 A & C). Four of the other transcription factor genes showed expression patterns similar to PRP2 (Figure S15 A, C, E & H). Interestingly, there were no significantly differentially expressed transcription factor genes found in common at the 50–100 mg or 400–500 mg seed weight stages in both backgrounds.

Table S2 contains the data for transcription factors that were differentially expressed between standard and defective seed coats in only the Clark or the Harsoy background. At the 50–100 mg seed weight stage, there were 59 transcription factor genes that showed differential expression in the seeds coats of only the Clark isolines with the maximum number of genes being related to the zinc finger category (Figure S16) whereas in the Harosoy isolines at the 50–100 mg seed weight stage, Glyma15g42810.1, was the only transcription factor gene that showed differential expression. Likewise, Figure S17 delineates the functional annotations of the 16 and 12 transcription factors that showed differential expression in the Clark background at the 100–200 or 400–500 mg stages, respectively, and Figure S18 shows the 43 and 111 transcription factors that were differentially expressed only in the Harosoy line at the later stages of development.

## Discussion

### Many Cell Wall Related Proteins are Affected by the Net Pattern Defective Seed Coat Mutation

We provide an overview of the genes that are affected by the “net pattern” mutation which affects the structural integrity of the soybean seed coat. As shown in Table 2 and Table S3, a total of 364 genes showed significant differential expression in both Clark and Harosoy isolines that contain the defective seed coat mutation. Approximately 15% of the differentially expressed genes showed cell wall related annotations including proline-rich proteins, glycine-rich proteins, fasciclin-like arabinogalactan proteins, expansins, and extensins. As reported earlier, these are the major classes of proteins that provide tensile strength and structure to the cell wall [19], [20]. Xyloglucan [21], xylulose [22] and pectin had also been reported as structural components of the cell wall [23], [24], [25], [26]. Structural cell wall GRPs have glycine contents up to 60–70% [27], [28], [29], [30]. In our study, genes with glycine-rich protein annotation had glycine content from 11–40% in their amino acid sequence.

Allergens, thaumatin-like proteins, and cysteine-rich domain containing proteins have also been reported to play important roles in various functions related to the cell wall [31], [32], [33]. The genes with these annotations were differentially expressed in standard and defective isolines in our study and may be involved in the appearance of cracking on the seed coat. Xylem serine proteinase1 was one of the significantly differentially expressed genes at the 100–200 mg seed weight stage in this study. In Arabidopsis, this enzyme has been reported to be involved in xylem formation [34].

While we have concentrated on the 51 cell wall related genes that were significantly differentially expressed in both varieties, we note that there are 920 additional cell wall related genes that did not show differential expression in the seed coats of either of the two isoline pairs. Thus, there are many processes related to the cell wall that are not affected at the transcript level by the net-pattern mutation.

### Hormone Regulated Genes Affected by the Net Pattern Mutation

Auxin is a plant growth regulator that is required for cell expansion, division and differentiation. Auxin pathway related genes have been reported to regulate several cell wall related genes in Arabidopsis [35], [36]. Likewise gibberellins had been reported to alter the expression of cell wall loosening genes and to work in coordination with auxin and abscisic acid hormone pathways [37]. Genes related to the jasmonic acid pathway and ethylene response may also be related to cell wall formation [38], [39]. As presented in the results (Tables S2–S4), there were differentially expressed genes in the standard and defective isolines that showed ethylene response related, auxin and gibberline hormone related/regulated annotations suggesting an effect of the seed coat mutation on these genes. The role of the auxin downregulated ADR12 transcript that showed high differential expression in the standard and defective seed coats but not in the hypocotyls (Figure 5) is not known. The predicted peptide form the ADR12 transcript is small at only 71 amino acids. Likely, many of the transcript differences for the affected pathways are confined to the seed coats as is the case for the ADR12 transcript. Such an extensive disruption of cell wall metabolism by the suite of genes affected in the net pattern mutation that leads to producing tears in the walls would likely be detrimental to the growth of the seedlings but is tolerated in the latter stages of seed coat development.

### Transcript Abundance and Levels of Soluble Proline-Rich Proteins are Reduced in the Defective Seed Coat Mutation

The proline-rich proteins are composed of small tandem repeats such as PPVYK or PPVEK, where the second proline is often hydroxyproline [40]. In our study PRP1 and PRP2 were among the highly differentially expressed genes (Tables 3 and 4). PRP1 was previously identified and characterized based on cDNA and amino acid sequence [41], [42], [43], [44]. Likewise, PRP2 had also been characterized as a slightly smaller protein than PRP1, and both consist essentially of the repeating decamer PPVYKPPVEK [45]. Gene model numbers corresponding to these genes were obtained from the Phytozome database that has genomic sequence from the Williams cultivar of *Glycine max* [46]. The Williams PRP2 protein is smaller when compared with the PRP2 in the cultivar Wayne from which it was originally sequenced as presented by amino acid sequence alignment in Figure S4. This corresponds with another study showing that in some soybean varieties both PRP1 and PRP2 proteins are smaller because of in frame deletions in the coding region of units of the tandem decamer repeats. In previous reports, PRP1 and PRP2 had also been reported to be linked, but separated by approximately 13% recombination units [47]. A BLAST search for these genes in Phytozome showed that they are located on chromosome 9, approximately 146 kb apart (Figure S5).

As described in our results, the expression of PRP1 was dramatically higher in the yellow Harosoy isolines (homozygous for *I* alleles), as compared to black Clark isolines (homozygous for the *i* allele) (Figure 4). Likewise, another study showed that the *I* locus which controls inhibition of anthocyanin accumulation in the epidermal cells of the soybean seed coat also affects abundance of PRP1 mRNA and protein in the seed coat. Interestingly, an epistatic interaction between the recessive *i* and *t* alleles also causes cracking of the pigmented seed coat [48]. This seed coat cracking is not related to the defective seed coats of the net pattern described in this report which is independent of seed color as shown in Figure 1. However, this genetic interaction implies an interaction of the flavonoid pathway with cell wall structure as the *t* locus is known to encode a flavonoid 3′ hydroxylase [17].

Our RNA-Seq data (Figure 4) show excellent agreement with the previous RNA blots [10] indicating a delay in the decline of PRP1 transcripts in the defective seed coats leading to higher levels in the defective seed coats at the middle weight range of 100–200 mg. Despite the presence of significant levels of PRP1 transcripts in the defective seed coats, no PRP1 protein was detectable by immunoblotting in defective Clark seed coats at any stage of seed development but it was easily extractable from the standard, non-defective isoline at the same stages [10]. However, the similar PRP2 protein was extractable from both standard and defective seed coats. These results implied that a major physiological event in the net pattern defective seed coats may be the irreversible cross-linking of PRP1 into the cell wall occurring in the developing seeds.

### Transcription Factors Affected by the Net Pattern Mutation

Transcription factors are important players for controlling the flow of genetic information from DNA to RNA and ultimately affecting the growth and physiology of the plant. In this study, there were approximately 240 differentially expressed transcription factor genes at different seed weight stages (Tables S2–S4). These genes were divided into different classes as presented in Figures 7, S16, S17, and S18. The major classes of transcription factors genes which showed differential expression in our study were Myb, NAC, basic helix-loop-helix, WRKY, and zinc finger transcription factors. Transcription factors of these types been reported to affect cell wall integrity in other plant species. MYB domain containing transcription factors are involved in secondary cell wall biosynthesis, pollen wall composition, mucilage deposition, extrusion and lignin deposition in Arabidopsis [49], [50], [51], [52]. The MYB46 and MYB83 transcription factors are thought to regulate secondary wall biosynthesis in Arabidopsis [53], [54]. A basic helix-loop-helix transcription factor had been reported to play an important role in tapetal cell development [55]. The role of a MADS-box transcription factor as RIPENING INHIBITOR (RIN) is in cell wall metabolism and carotenoid biosynthesis [56] and a basic leucine zipper domain containing transcription factor had been reported to affect pollen coat patterning [57]. As demonstrated by Wang *et al.,* WRKY transcription factors are involved in regulation of downstream genes encoding the NAM, ATAF1/2, and CUC2 (NAC) and CCCH type (C3H) zinc finger TFs that activate secondary wall synthesis [58]. NAC domain containing transcription factors were reported to be involved in secondary cell wall biosynthesis and in the regulation of cellulose and hemicellulose biosynthetic genes in addition to those involved in lignin polymerization and signaling in Arabidopsis and Eucalyptus [59], [60]. Rice and maize SWNs (Secondary wall NACs) and MYB46 had been reported as master transcriptional activators of the secondary wall biosynthetic program [61]. The AP2 domain containing family is an ethylene responsive group of transcription factors, and in *Arabidopsis thaliana*, these were expressed in specific cell types of roots, stems and seeds that undergo suberization [62]. The TT1 genes were another class of the significantly differentially expressed genes in this study (Figure 6). These transcription factor genes had been reported to be involved in seed coat pigmentation and integrity in Arabidopsis [63].

It is likely that the defective seed coat mutation in soybean sets in motion changes in many pathways related to the cell wall. Some of the differential expression may be a temporal shift in developmental appearances of transcripts. Notably, in the case of the TT1 homolog, it appears that the decline in its transcript levels was delayed in the defective seed coats relative to the standard seed coats (Figure 6), thus leading to significant differential expression at the 100–200 mg seed weight range. This profile is similar to the pattern exhibited by the PRP1 protein (Figure 4A) in that its decline in expression appears to be slowed. Thus, a developmental delay in the normal expression patterns of multiple genes appeared to be a feature of the effect of this mutation as shown in Figures S6–S10 and S14–S15. These shifts may be one of the reasons for significant differential expression at the mid-seed weight range of 100–200 mg and may indicate that the triggering event occurs at or before the 50 mg seed weight range.

### Summary

We present an overview of genes whose expression was affected by the “net pattern” mutation in soybean. Two isolines of different genetic backgrounds were used to understand physiology related to this mutation that causes defective cracks in the seed coats. Twelve samples representing three stages of seed coat development from each of the four lines were subjected to the power of next-generation sequencing to obtain millions of transcript reads that were mapped to all 78,773 high and low confidence gene models of *Glycine max*. There were approximately 1300 significantly differentially expressed genes affected by this mutation in each isoline pair with 364 in common between the two isolines of different genetic backgrounds. The cell wall structural protein genes including proline-rich proteins (PRP1 and PRP2) and glycine-rich proteins were among the transcripts affected by the mutation as well as a number of transcription factors. Some of the transcript patterns indicate that a developmental shift in timing of gene expression underpins the differential gene expression changes at mid-maturation as shown by graphical presentation of expression patterns for 82 out of 364 genes that were significantly differentially expressed in both the Clark and Harosoy backgrounds. In summary, the mutation appears to set in motion a complex series of events, many manifested at the transcript level, that lead to changes in physiology and ultimately structure of the cell wall. The information on potential candidate genes for this mutation and the regulated genes will not only be helpful in understanding cell wall physiology in soybean, but could also provide a platform for improvement in bioenergy crops like sorghum, maize and *Miscanthus*.

## Methods

### Plant Materials and Genetic Nomenclature

The isolines of *Glycine max* used in this study were a black seeded Clark line, Clark defective seed coat, Harosoy standard and Harosoy defective seed coat. The defective seed coat mutation was originally found in two PI (plant introduction) lines, PI 339.994 and PI423.730B and these lines were backcrossed to the black seeded Clark line (UC9) to create an isoline UC412 containing this mutation. The same mutation was also backcrossed into the Harosoy line to create an isoline UC508. The L or PI numbers represent the official USDA isoline or plant introduction numbers, respectively. The UC number is an internal number used by our laboratory. Detailed information on these lines is presented in Table 1.

### RNA-Seq Method

For RNA extraction, immature seeds were harvested over the course of several weeks. The individual seeds were pooled and sorted by weight. Following this, each seed was dissected, separating the cotyledon and embryo from the seed coat, and placed in separate 15-ml polypropylene centrifuge tubes (Corning, Acton, MA). All tissue was then frozen in liquid nitrogen and placed in storage at −80°C until it could be lyophilized. RNA of seed coats of seeds from the 50–100 mg, 100–200 mg and 400–500 mg seed weight stages of each isoline was extracted separately using the RNA for 5 ml volumes from ∼30 mg (50–100 mg and 100–200 mg seed weight stage) and ∼70 mg (400–500 mg seed weight stage) dry weight. The modified protocol used here is based on the protocols of McCarty [64]. Extraction of RNA from seed coats with dark pigmentation requires the use of a modified extraction protocol which prevents procyanidins from binding the RNA [65], [66]. RNA was extracted from 200 mg of freeze-dried seed coats by using phenol chloroform extraction and lithium chloride precipitation supplemented with PVPP (polyvinylpyrrolidone), polyproline and BSA (bovine serum albumin). Library construction and high-throughput sequencing was carried out using RNA-Seq technology using Illumina GaII and HiSeq2000 instruments by Keck Center, University of Illinois.

### RNA-Seq Alignment and Data Normalization

The 75 bp and 100bp reads were mapped to the 78,773 Glyma cDNA gene models (JGI/Phytozome) using Bowtie [11] with up to 3 mismatches allowed and up to 25 alignments. The total number of generated raw reads for Clark standard and defective isolines and for Harosoy isolines at three different stages of seed development is presented in Table 1. RNA-Seq data were normalized in reads per kilobase of gene model per million mapped reads (RPKM), as the RNA-Seq depends on the transcript length since the reads will map to all positions of the transcript [18].

#### Annotation of glyma gene models

Coding region gene models were collected from the masked soybean genome from Phytozome version 6.0 and the soybean genome version 1.0 GFF file of the soybean genome [12] which is available at SoyBase [67]. In addition to the PFAM annotations that were downloaded from Phytozome, the 78,773 models (that include both high and low confidence models) were further annotated using BLASTX against the non-redundant (nr) database of the National Center for Biotechnology Information (NCBI), and for trEMBL and Swiss prot of the European Bioinformatics Institute (EBI) as described by Hunt *et al*. [68].

#### DESeq data analysis

DESeq (available via Bioconductor) is an R package to analyze count data from high-throughput sequencing data such as RNA-Seq and test for differential expression [69]. The basemean value and p values for each Glyma model were analyzed using this package for 78,773 Glyma models using comparison between Clark standard and defective or Harosoy standard and defective isolines.

### Northern Blots

Total RNA was extracted from the frozen tissue including 4–5 day old soybean hypocotyls minus their cotyledons and seed coats of 100–200 mg seeds from four lines using a standard phenol chloroform method with lithium chloride precipitation [64] or the modified method for pigmented tissues [65], [66]. RNA samples were quantified by spectrophotometer and the integrity was confirmed using agarose gel electrophoresis. RNA was stored at −80°C until further use. For RNA gel blot analysis, 10 µg of total RNA was electrophoresed through 1.2% agarose/1.1% formaldehyde gels [70] blotted onto nitrocellulose membranes (Schleicher & Schuell, Keene, NH) via capillary action with 10× SSC (1.5 M NaCl and 0.15 M sodium citrate, pH = 7) overnight. After blotting, RNA was cross-linked to the nitrocellulose membranes with UV radiation by a UV cross-linker (Stratagene, La Jolla, CA). Nitrocellulose RNA gel blots were then prehybridized, hybridized, washed, and exposed to Hyperfilm (Amersham, Piscataway,NJ) as described by Todd and Vodkin [14]. An ADR12 gene EST was (Gm-r1088-6964) used as probe and labeled with [α-^32^P]dATP by random primer reaction method [71].

### Accession Numbers

The data have been entered into Gene Expression Omnibus at that National Center for Biotechnology Information as Accession Series GSE54903.

## Supporting Information

Figure S1 The Distribution of Expression Levels in RPKMs of Differentially Expressed Genes in the Clark Isoline Pair at Three Different Seed Weight Stages. (A) 50–100 mg in Clark Standard (B) 50–100 mg in Clark Defective (C) 100–200 mg in Clark Standard (D) 100–200 mg in Clark Defective (E) 400–500 mg Clark Standard (F) 400–500 mg Clark Defective.
(TIFF)

Figure S2 The Distribution of Expression Levels of Differentially Expressed Genes in the Harosoy Isoline Pair at Three Different Seed Weight Stages. (A) 50–100 mg in Harosy Standard (B) 50–100 mg in Harosoy Defective (C) 100–200 mg in Harosoy Standard (D) 100–200 mg in Harosoy Defective (E) 400–500 mg Harosoy Standard (F) 400–500 mg Harosoy Defective.
(TIFF)

Figure S3 The Nucleotide and Amino Acid Sequence Alignment for PRP1. (A) Alignment of PRP1 nucleotide sequence from NCBI (J02746) and PRP1 sequence (Glyma09g12200.1) from Phytozome database (B) Alignment of PRP1 amino acid sequence from NCBI with PRP1 amino acid sequence from Phytozome database. The nucleotide and amino acid sequence from *Williams* (Phytozome) and *Wayne* (NCBI) cultivars showed 100% sequence homology.
(TIFF)

Figure S4 The Nucleotide and Amino Acid Sequence Alignment of the PRP2 Gene. (A) Alignment of PRP2 nucleotide sequence from NCBI (J05208) and PRP2 sequence from Phytozome database (Glyma09g12260.1) (B) Alignment of PRP2 amino acid sequence from NCBI with PRP2 amino acid sequence from Phytozome database. There is difference of one tandem repeat in the amino acid sequence. The PRP2 protein is shorter in Williams (Phytozome) as compared to Wayne (NCBI) cultivar.
(TIFF)

Figure S5 The Genome Organization of PRP1 and PRP2. As in the Phytozome database, PRP1 and PRP2 are on chromosome 9 and the distance between these genes is ∼146 kb.
(TIFF)

Figure S6 The Expression Pattern of 13 Selected Differentially Expressed Genes in the Seed Coat of Wildtype and Defective Isolines in Both Clark and Harosoy Backgrounds. The genes over expressed in the defective isolines (Tables 3a, 4a and Table S3). CS: Clark Standard, CD: Clark Defective, HS: Harosoy Standard, HD: Harosoy Defective.
(TIF)

Figure S7 The Expression Pattern of 16 Selected Differentially Expressed Genes in the Seed Coat of Wildtype and Defective Isolines in Both Clark and Harosoy Background. The genes overexpressed in the Standard isolines (Tables 3b, 4b and Table S3). CS: Clark Standard, CD: Clark Defective, HS: Harosoy Standard, HD: Harosoy Defective.
(TIF)

Figure S8 The Expression Pattern of 11 Fasciclin-like Arabinogalactan Gene in the Seed Coat of Wildtype and Defective Isolines in Both Clark and Harosoy Background. These genes showed higher expression in defective isoline as compare to wildtype isoline (Table S3). CS: Clark Standard, CD: Clark Defective, HS: Harosoy Standard, HD: Harosoy Defective.
(TIF)

Figure S9 The Expression Pattern of Cell Wall Genes Overexpressed in the Seed Coats of Defective Isolines in Both Clark and Harosoy Background. These graphs present data for 25 additional cell wall related genes not previously shown in Figure S6 and S7. Their RPKM and p value data are presented in Table S3. CS: Clark Standard, CD: Clark Defective, HS: Harosoy Standard, HD: Harosoy Defective.
(TIF)

Figure S10 The Expression Pattern of Cell Wall Genes Overexpressed in the Standard Seed Coats of Both Clark and Harosoy Background. These graphs present data for 3 additional cell wall related genes not previously shown in Figure S6 and S7. Their RPKM and p value data are presented in Table S3. CS: Clark Standard, CD: Clark Defective, HS: Harosoy Standard, HD: Harosoy Defective.
(TIFF)

Figure S11 The Distribution of Differentially Expressed Genes in the Seed Coats of Either (A) Clark or (B) Harosoy Backgrounds at the 50–100 mg Seed Weight Stage. The number of differentially expressed genes (≥5RPKM, ≥2 fold differential expression, p-value≤0.05) was (A) 720 genes in Clark isolines and (B) 48 genes in Harosoy isolines. In both backgrounds, there were major categories related to the cell wall.
(TIFF)

Figure S12 The Distribution of Differentially Expressed Genes in Seed Coats of Either (A) Clark or (B) Harosoy Backgrounds at the 100–200 mg Seed Weight Stage. The number of differentially expressed genes (≥5RPKM, ≥2 fold differential expression, p-value ≤0.05) was (A) 173 genes in Clark isolines and (B) 156 genes in Harosoy isolines at 100–200 mg seed weight stage. In both backgrounds, one of the major categories was related to the cell wall.
(TIFF)

Figure S13 The Distribution of Differentially Expressed Genes in Seed Coats of (A) Clark or (B) Harosoy Backgrounds at the 400–500 mg Seed Weight Stage. The number of differentially expressed genes (≥5RPKM, ≥2 fold differential expression, p-value ≤0.05) was (A) 417 genes in Clark isolines and (B) 1068 genes in Harosoy isolines. In both backgrounds, the cell wall related genes occupied 5% of the chart area.
(TIFF)

Figure S14 The Expression Pattern of Differentially Expressed Transcription Factor Genes That Showed Higher Expression in the Seed Coat of Defective Isolines in Both Clark and Harosoy Background. Overexpressed in defective isolines. CS: Clark Standard, CD: Clark Defective, HS: Harosoy Standard, HD: Harosoy Defective.
(TIF)

Figure S15 The Expression Pattern of Differentially Expressed Transcription Factor Genes That Showed Higher Expression in the Seed Coat of Standard Isolines in Both Clark and Harosoy Background. Overexpressed in standard isolines. CS: Clark Standard, CD: Clark Defective, HS: Harosoy Standard, HD: Harosoy Defective.
(TIF)

Figure S16 The Distribution of Differentially Expressed Transcription Factor Genes into Different Classes in Clark at the 50–100 mg Seed Weight Stage. The 59 differentially expressed transcription factor genes (≥5RPKM, ≥2 fold differential expression, p-value ≤0.05) were divided into 12 classes based on functional annotations.
(TIFF)

Figure S17 The Distribution of Differentially Expressed Transcription Factor Genes in (A) Clark or (B) Harosoy Backgrounds at the 100–200 mg Seed Weight Stage. (A) The 16 differentially expressed transcription factor genes (≥5RPKM, ≥2 fold differential expression, p-value≤.05) were divided into 9 different classes (B) The 12 differentially expressed transcription factor genes (≥5RPKM, ≥2 fold differential expression, p-value ≤0.05) were divided into 10 different classes based on functional annotation.
(TIFF)

Figure S18 The Distribution of Differentially Expressed Transcription Factor Genes in Different Classes in (A) Clark and (B) Harosoy Backgrounds at the 400–500 mg Seed Weight Stage. (A) The 43 differentially expressed transcription factor genes (≥5RPKM, ≥2 fold differential expression, p-value ≤.05) were divided into 10 different classes (B) The 111 differentially expressed transcription factor genes (≥5RPKM, ≥2 fold differential expression, p-value ≤0.05) were divided into 15 different classes based on functional annotation.
(TIFF)

Table S1 The Total Number of Expressed Genes in Seed Coats of the Different Isolines. The total number of expressed genes at ≥10 RPKM or ≥1 RPKM in different backgrounds at different seed weight stages are presented in this table. CS: Clark standard, CD: Clark defective, HS: Harosoy Standard and HD: Harosoy defective.
(DOCX)

Table S2 Significantly Differentially Expressed Genes Found in the Seed Coats of Either Clark or Harosoy Backgrounds at the Indicated Stages of Seed Development.
(XLSX)

Table S3 The 364 Significantly Differentially Expressed Genes Found in Common in Both Clark and Harosoy Backgrounds as Defined in Table 2.
(XLSX)

Table S4 The 109 Significantly Differentially Expressed Genes Common in Both Background But with Expression in Opposite Directions as Described in Table 2.
(XLSX)
