# Supplementary figures and images for: RNA-Seq Profiling of a Defective Seed Coat Mutation in Glycine max Reveals Differential Expression of Proline-Rich and Other Cell Wall Protein Transcripts
Source: PLoS One. 2014 May 14;9(5):e96342. doi: 10.1371/journal.pone.0096342 (PMC4020777; doi:10.1371/journal.pone.0096342)

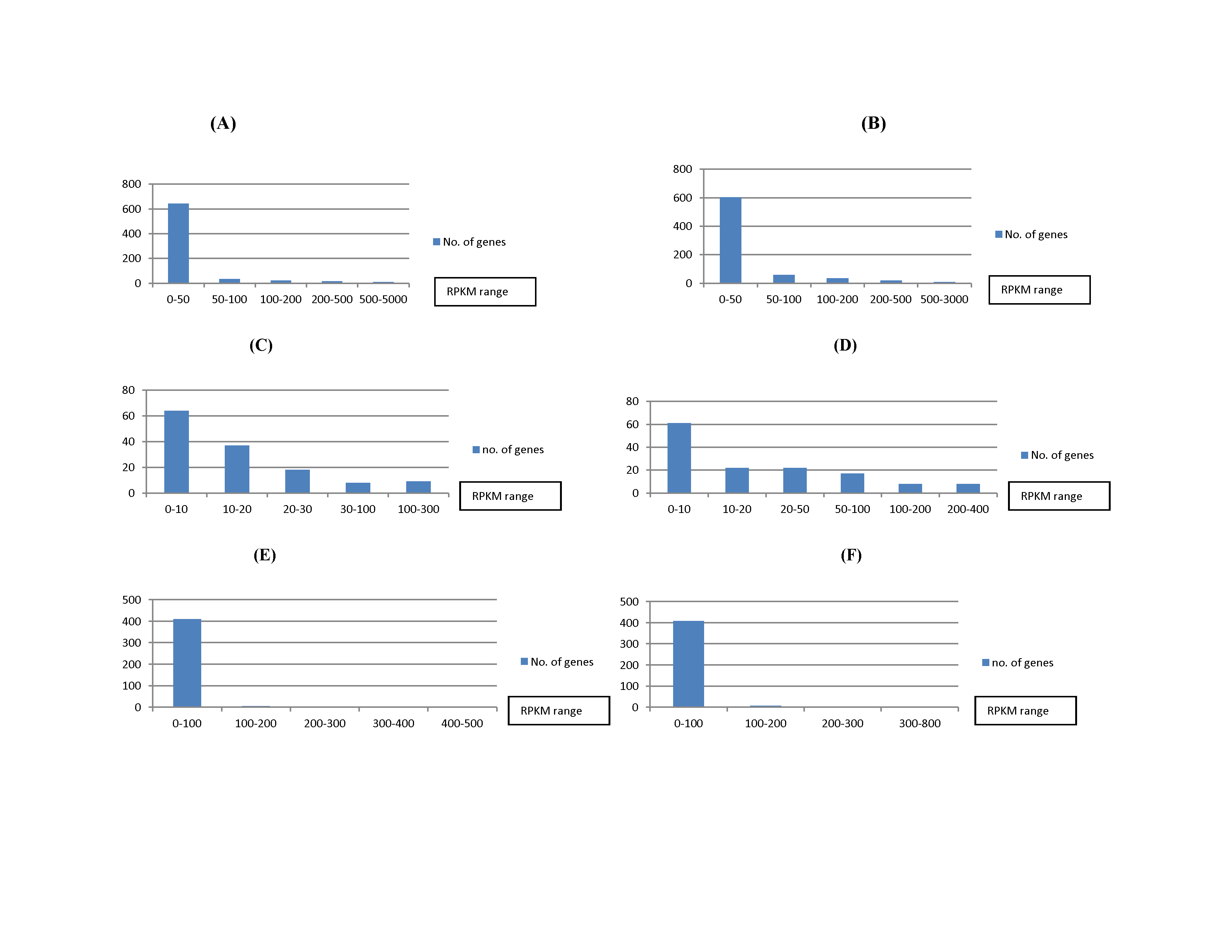

Supplement: Figure S1 — The Distribution of Expression Levels in RPKMs of Differentially Expressed Genes in the Clark Isoline Pair at Three Different Seed Weight Stages. (A) 50–100 mg in Clark Standard (B) 50–100 mg in Clark Defective (C) 100–200 mg in Clark Standard (D) 100–200 mg in Clark Defective (E) 400–500 mg Clark Standard (F) 400–500 mg Clark Defective. (TIFF) [file pone.0096342.s001.tiff]

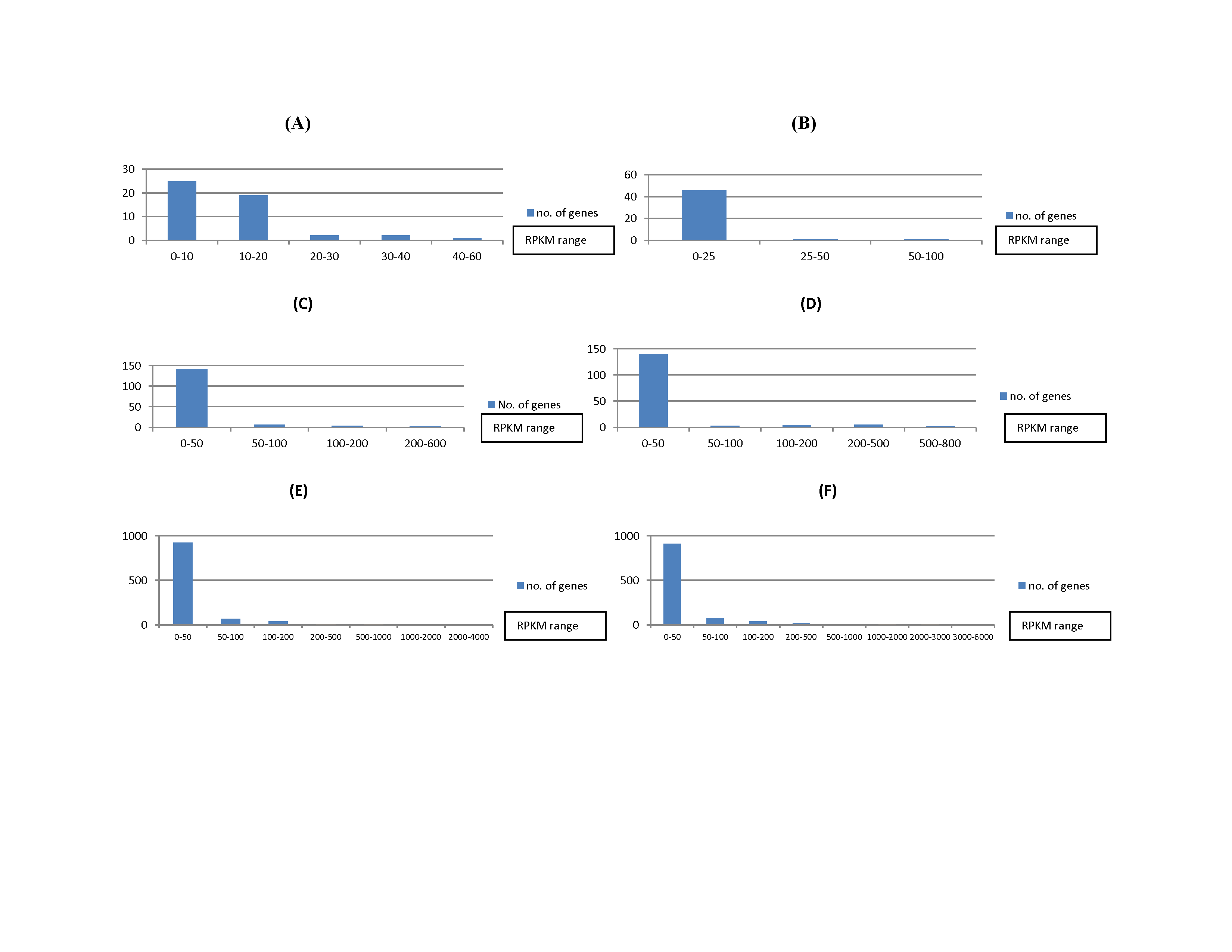

Supplement: Figure S2 — The Distribution of Expression Levels of Differentially Expressed Genes in the Harosoy Isoline Pair at Three Different Seed Weight Stages. (A) 50–100 mg in Harosy Standard (B) 50–100 mg in Harosoy Defective (C) 100–200 mg in Harosoy Standard (D) 100–200 mg in Harosoy Defective (E) 400–500 mg Harosoy Standard (F) 400–500 mg Harosoy Defective. (TIFF) [file pone.0096342.s002.tiff]

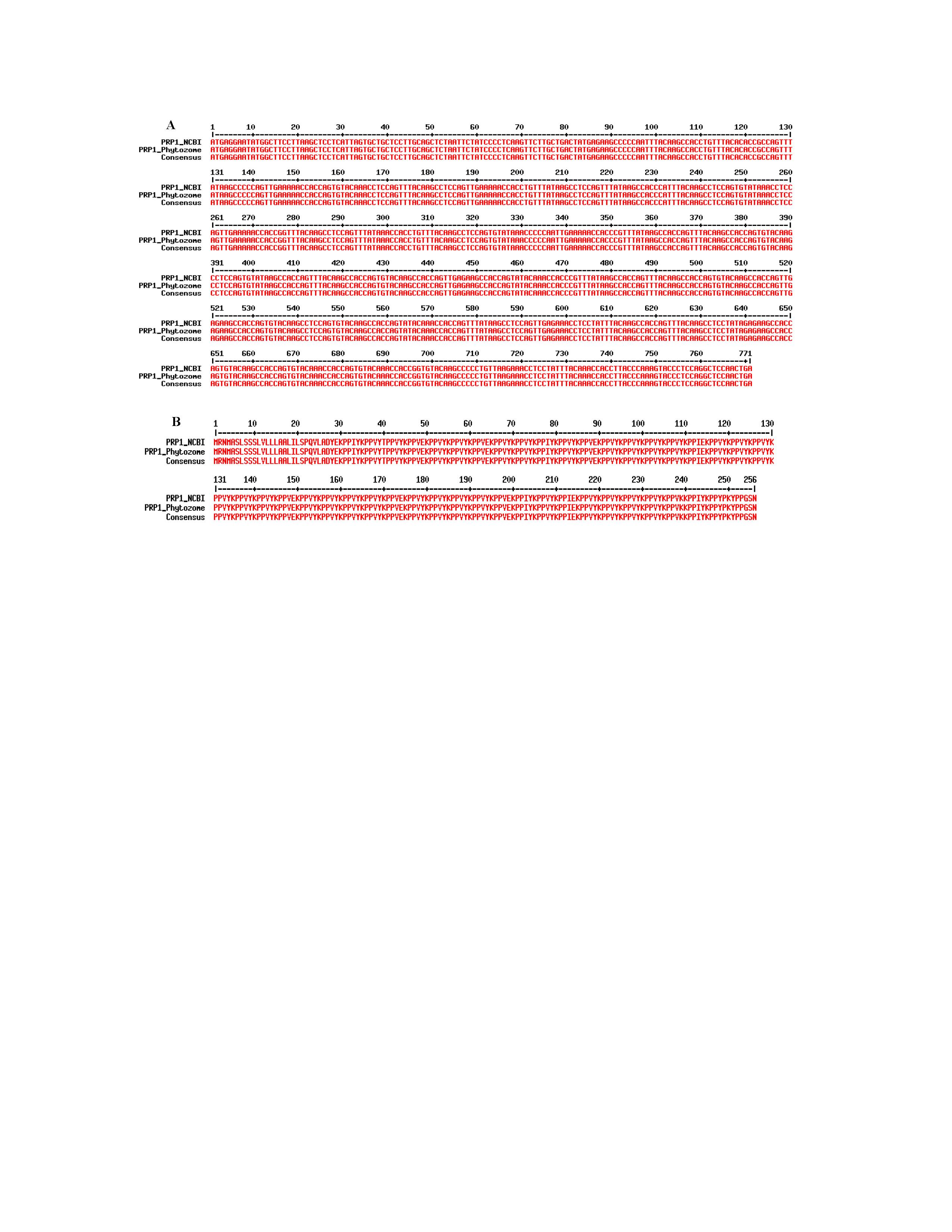

Supplement: Figure S3 — The Nucleotide and Amino Acid Sequence Alignment for PRP1. (A) Alignment of PRP1 nucleotide sequence from NCBI (J02746) and PRP1 sequence (Glyma09g12200.1) from Phytozome database (B) Alignment of PRP1 amino acid sequence from NCBI with PRP1 amino acid sequence from Phytozome database. The nucleotide and amino acid sequence from Williams (Phytozome) and Wayne (NCBI) cultivars showed 100% sequence homology. (TIFF) [file pone.0096342.s003.tiff]

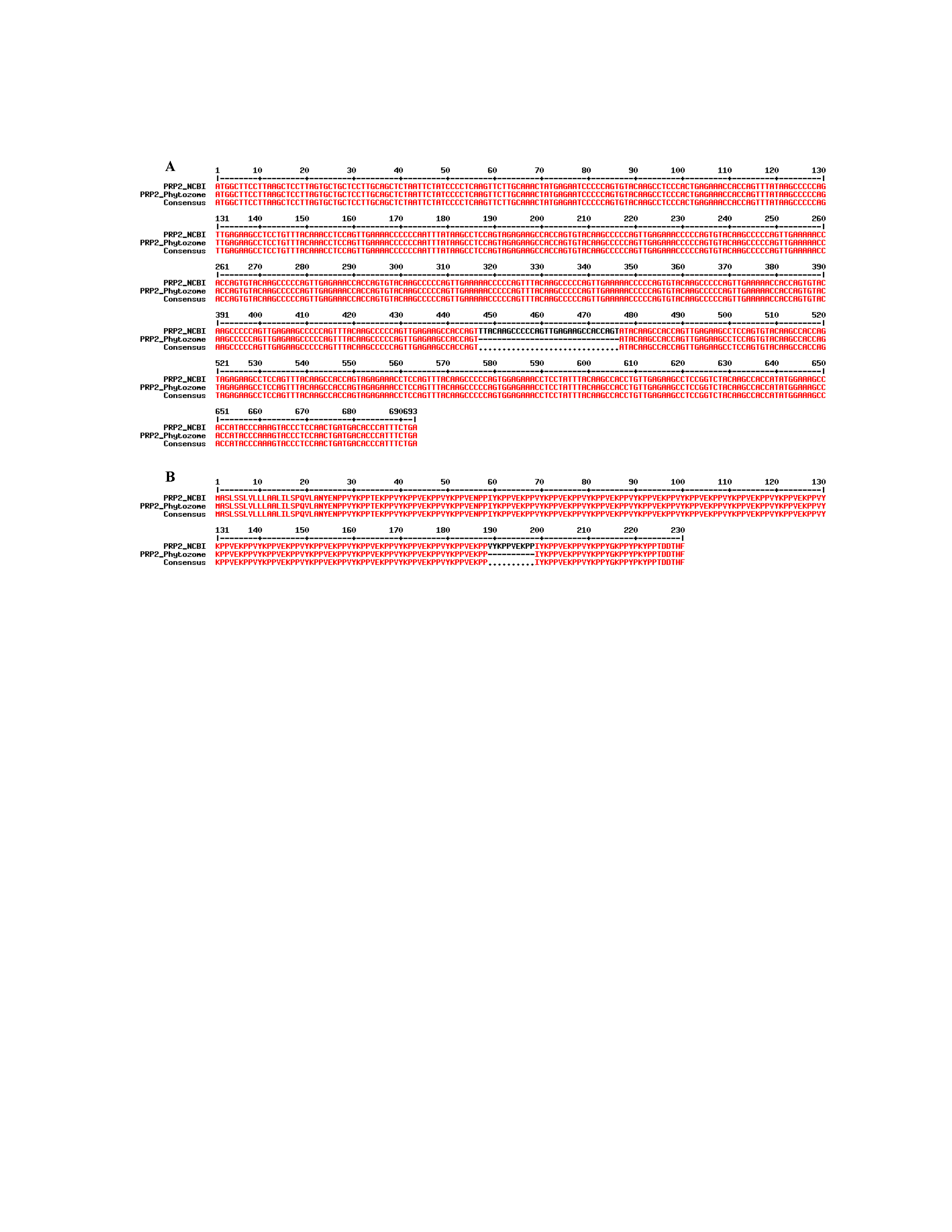

Supplement: Figure S4 — The Nucleotide and Amino Acid Sequence Alignment of the PRP2 Gene. (A) Alignment of PRP2 nucleotide sequence from NCBI (J05208) and PRP2 sequence from Phytozome database (Glyma09g12260.1) (B) Alignment of PRP2 amino acid sequence from NCBI with PRP2 amino acid sequence from Phytozome database. There is difference of one tandem repeat in the amino acid sequence. The PRP2 protein is shorter in Williams (Phytozome) as compared to Wayne (NCBI) cultivar. (TIFF) [file pone.0096342.s004.tiff]

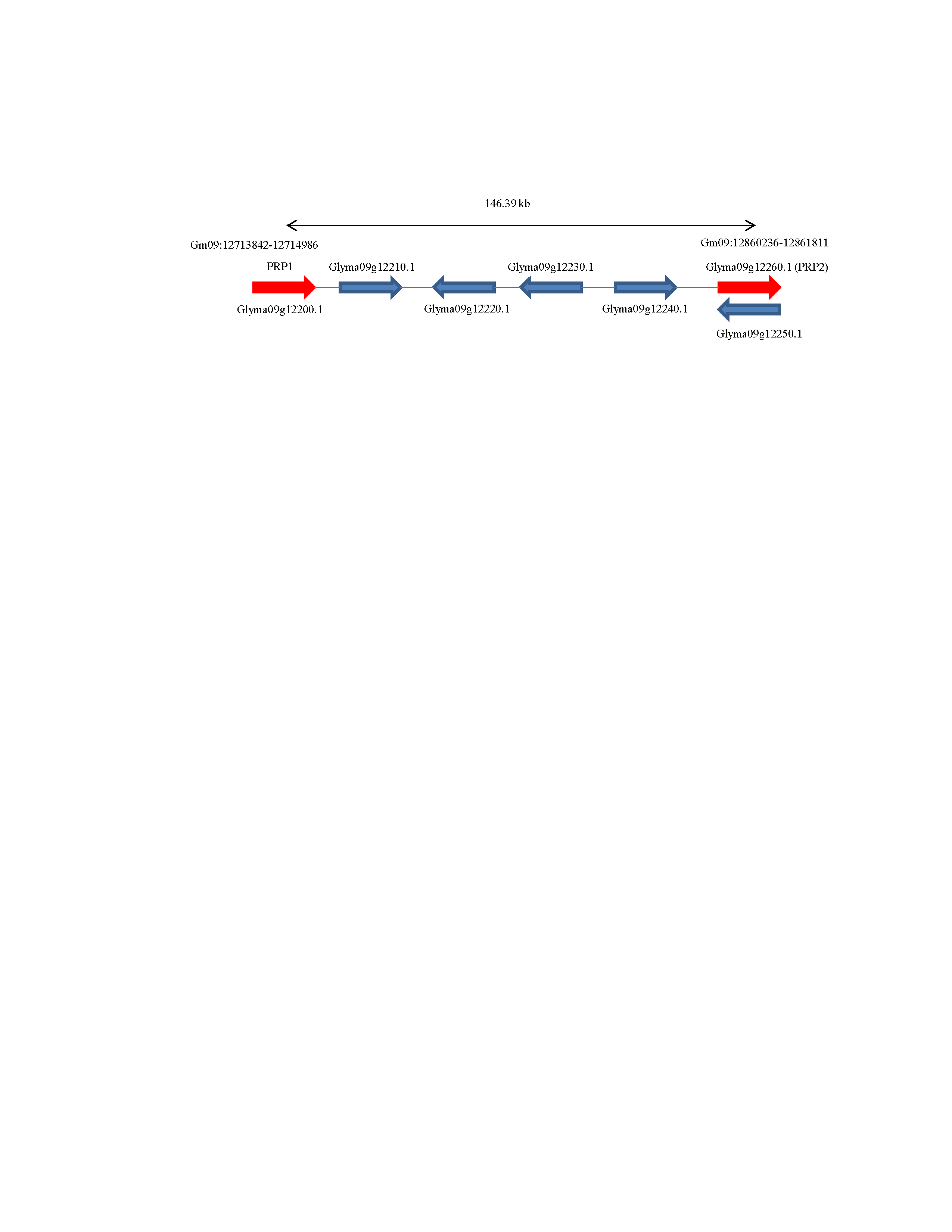

Supplement: Figure S5 — The Genome Organization of PRP1 and PRP2. As in the Phytozome database, PRP1 and PRP2 are on chromosome 9 and the distance between these genes is ∼146 kb. (TIFF) [file pone.0096342.s005.tiff]

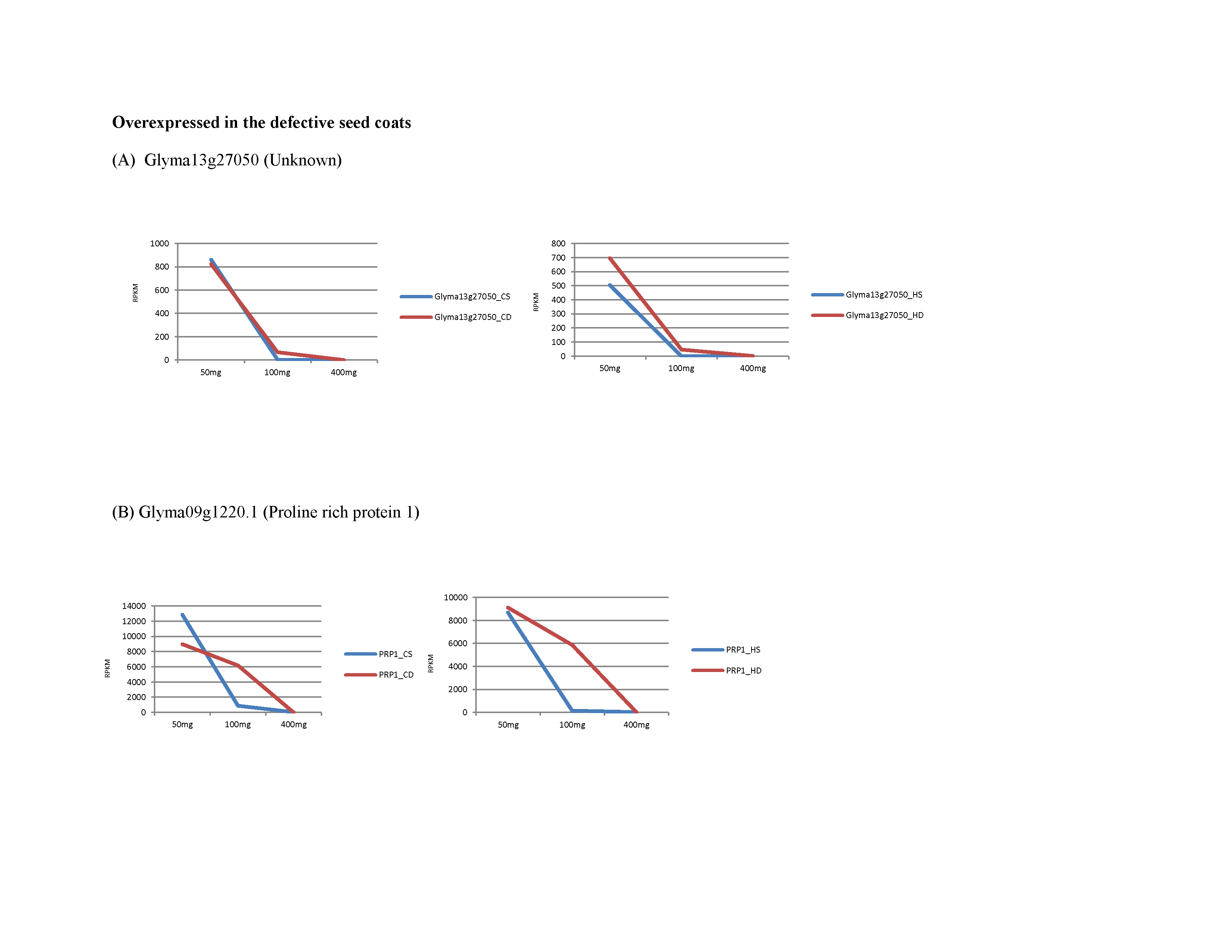

Supplement: Figure S6 — The Expression Pattern of 13 Selected Differentially Expressed Genes in the Seed Coat of Wildtype and Defective Isolines in Both Clark and Harosoy Backgrounds. The genes over expressed in the defective isolines (Tables 3a, 4a and Table S3). CS: Clark Standard, CD: Clark Defective, HS: Harosoy Standard, HD: Harosoy Defective. (TIF) [file pone.0096342.s006.tif]

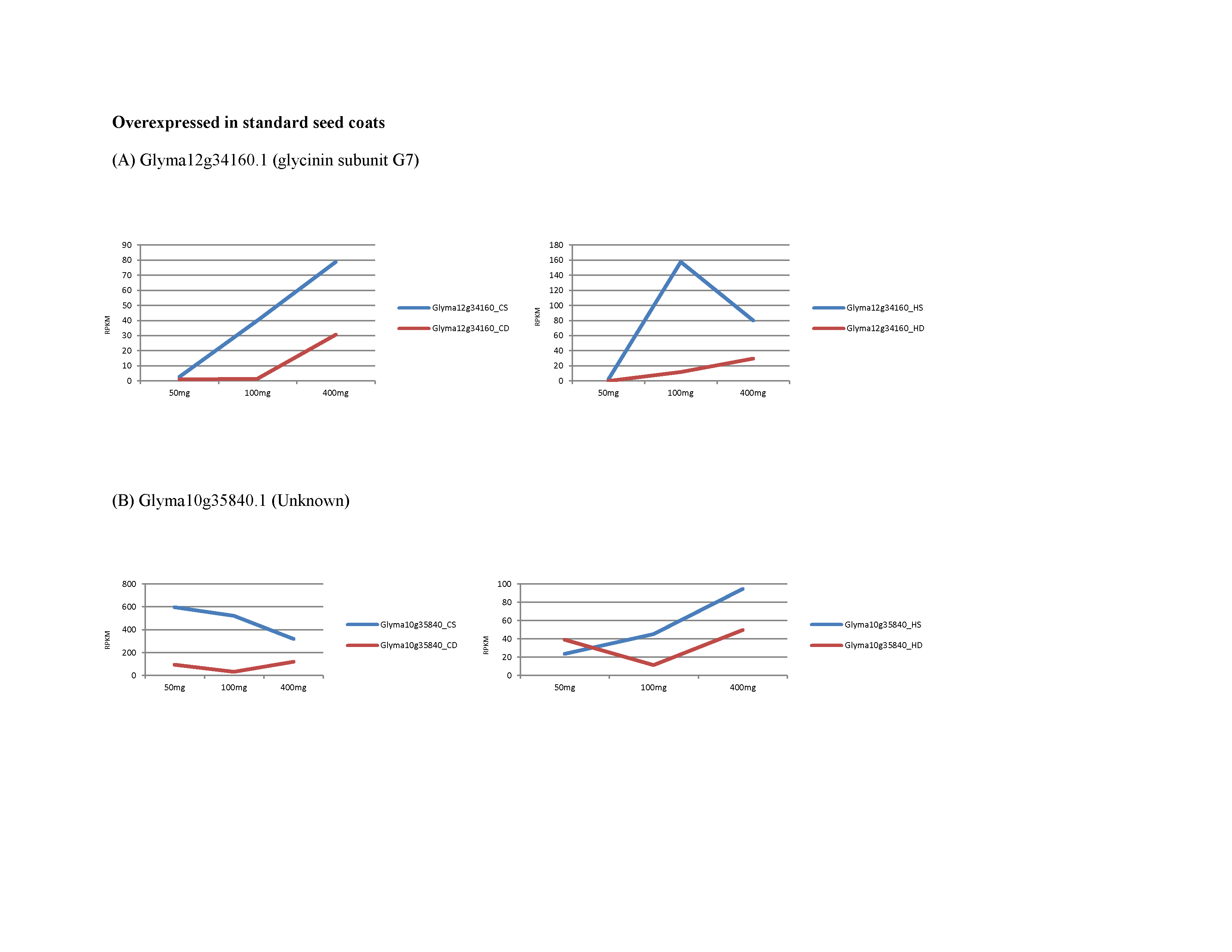

Supplement: Figure S7 — The Expression Pattern of 16 Selected Differentially Expressed Genes in the Seed Coat of Wildtype and Defective Isolines in Both Clark and Harosoy Background. The genes overexpressed in the Standard isolines (Tables 3b, 4b and Table S3). CS: Clark Standard, CD: Clark Defective, HS: Harosoy Standard, HD: Harosoy Defective. (TIF) [file pone.0096342.s007.tif]

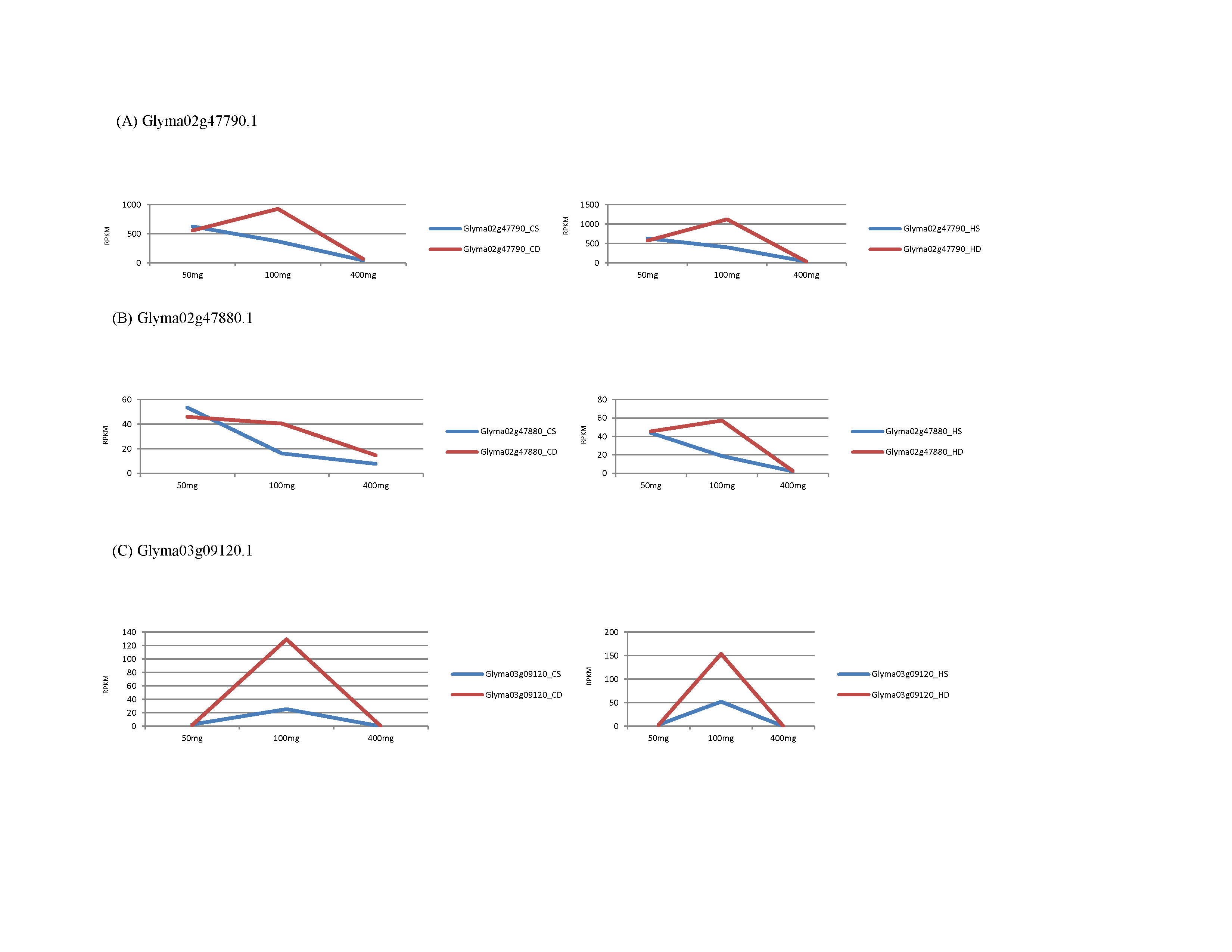

Supplement: Figure S8 — The Expression Pattern of 11 Fasciclin-like Arabinogalactan Gene in the Seed Coat of Wildtype and Defective Isolines in Both Clark and Harosoy Background. These genes showed higher expression in defective isoline as compare to wildtype isoline (Table S3). CS: Clark Standard, CD: Clark Defective, HS: Harosoy Standard, HD: Harosoy Defective. (TIF) [file pone.0096342.s008.tif]

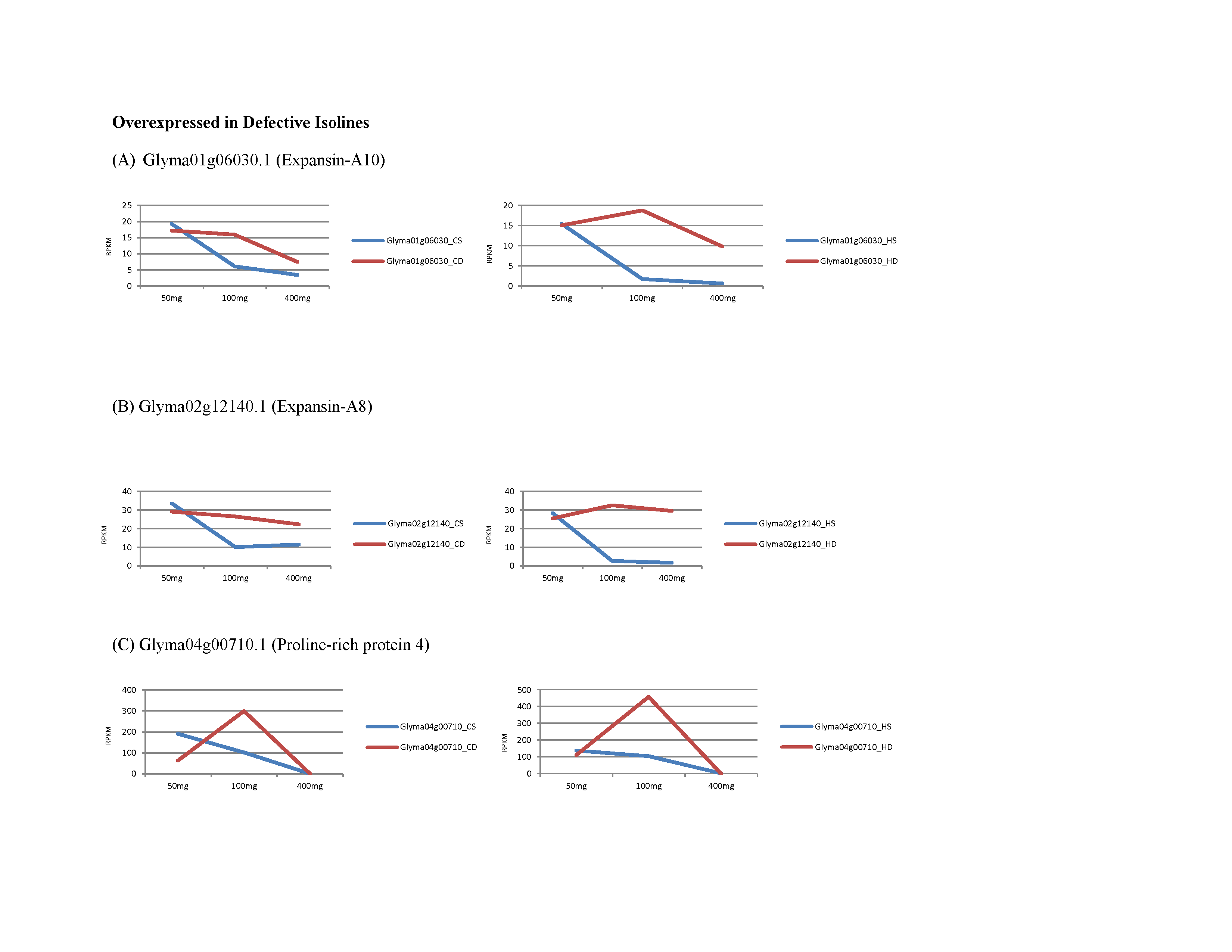

Supplement: Figure S9 — The Expression Pattern of Cell Wall Genes Overexpressed in the Seed Coats of Defective Isolines in Both Clark and Harosoy Background. These graphs present data for 25 additional cell wall related genes not previously shown in Figure S6 and S7. Their RPKM and p value data are presented in Table S3. CS: Clark Standard, CD: Clark Defective, HS: Harosoy Standard, HD: Harosoy Defective. (TIF) [file pone.0096342.s009.tif]

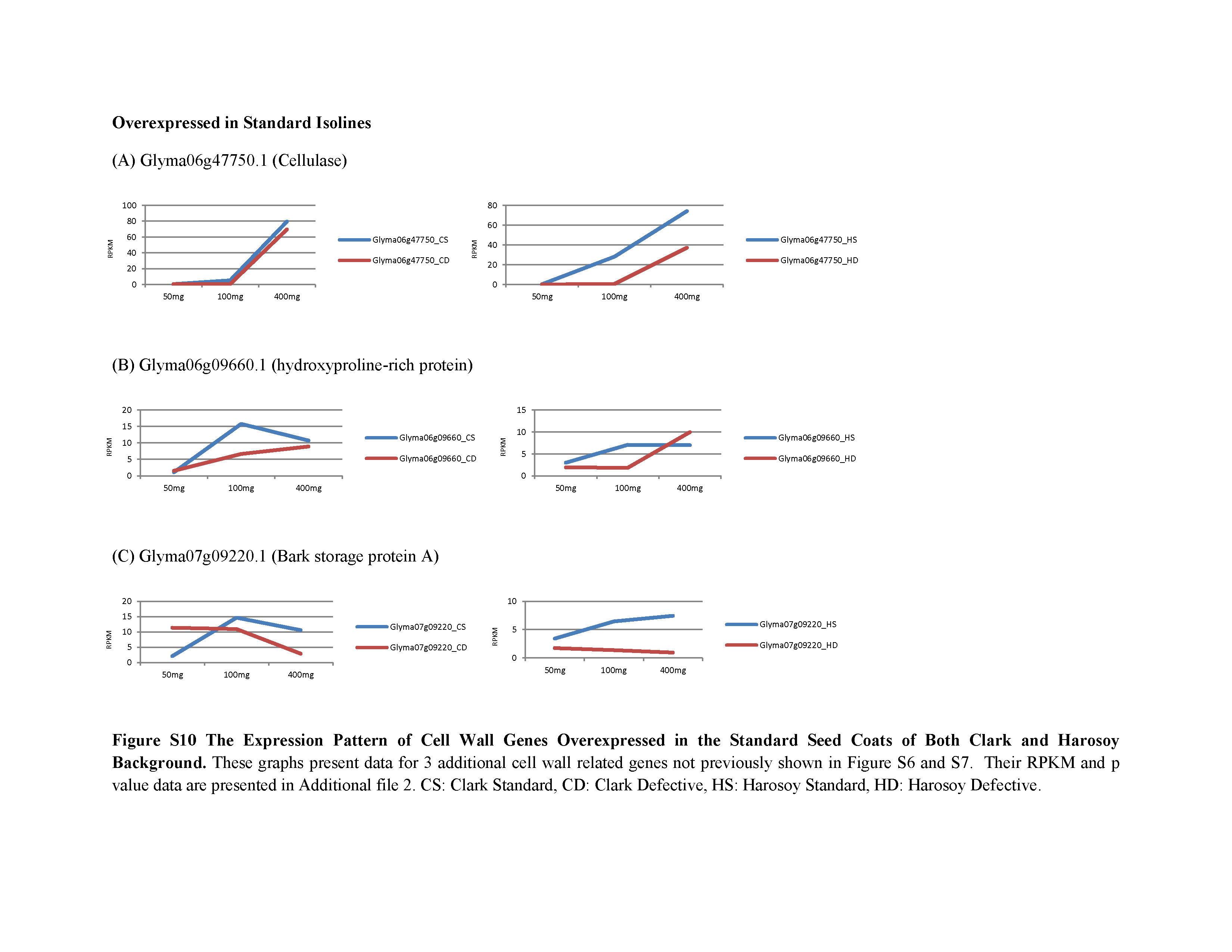

Supplement: Figure S10 — The Expression Pattern of Cell Wall Genes Overexpressed in the Standard Seed Coats of Both Clark and Harosoy Background. These graphs present data for 3 additional cell wall related genes not previously shown in Figure S6 and S7. Their RPKM and p value data are presented in Table S3. CS: Clark Standard, CD: Clark Defective, HS: Harosoy Standard, HD: Harosoy Defective. (TIFF) [file pone.0096342.s010.tiff]

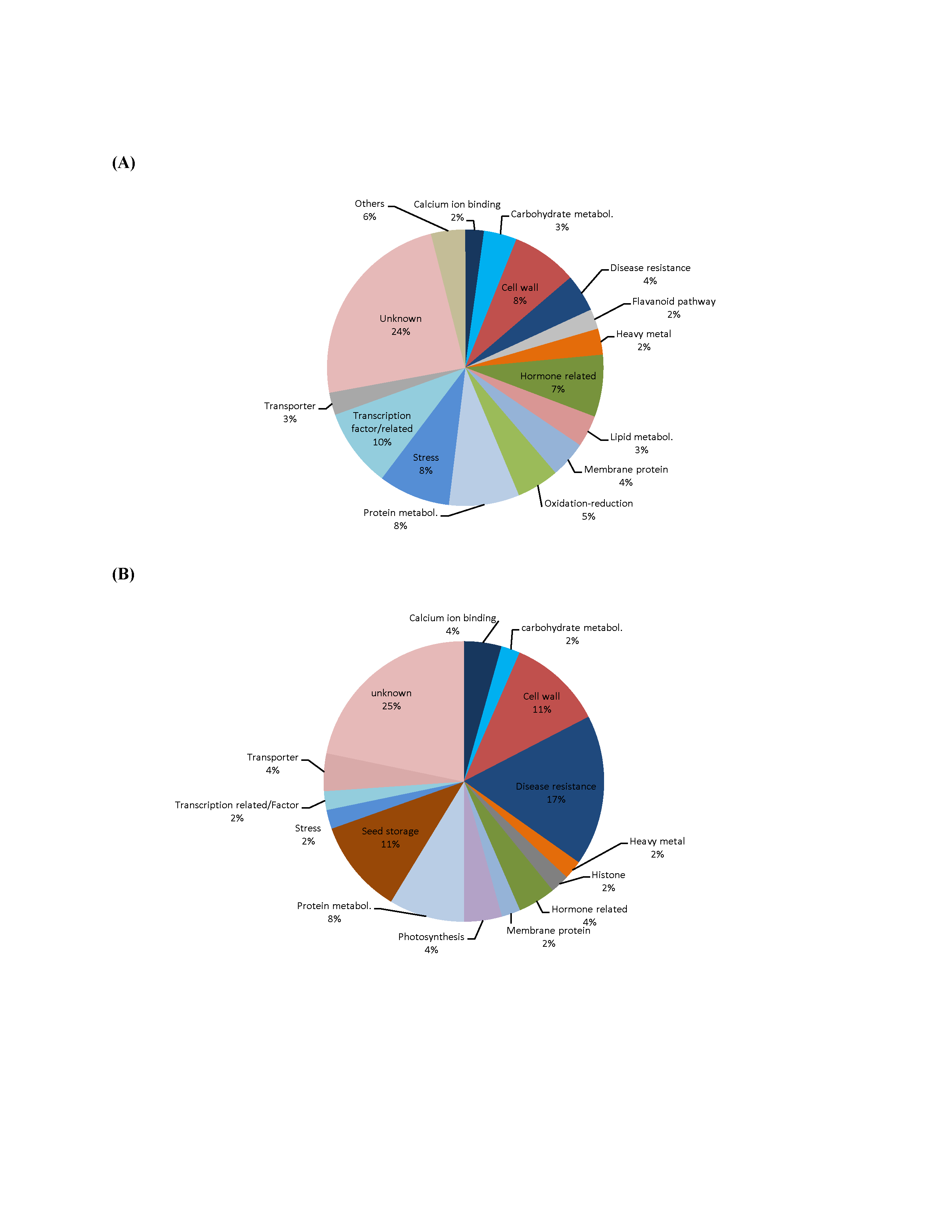

Supplement: Figure S11 — The Distribution of Differentially Expressed Genes in the Seed Coats of Either (A) Clark or (B) Harosoy Backgrounds at the 50–100 mg Seed Weight Stage. The number of differentially expressed genes (≥5RPKM, ≥2 fold differential expression, p-value≤0.05) was (A) 720 genes in Clark isolines and (B) 48 genes in Harosoy isolines. In both backgrounds, there were major categories related to the cell wall. (TIFF) [file pone.0096342.s011.tiff]

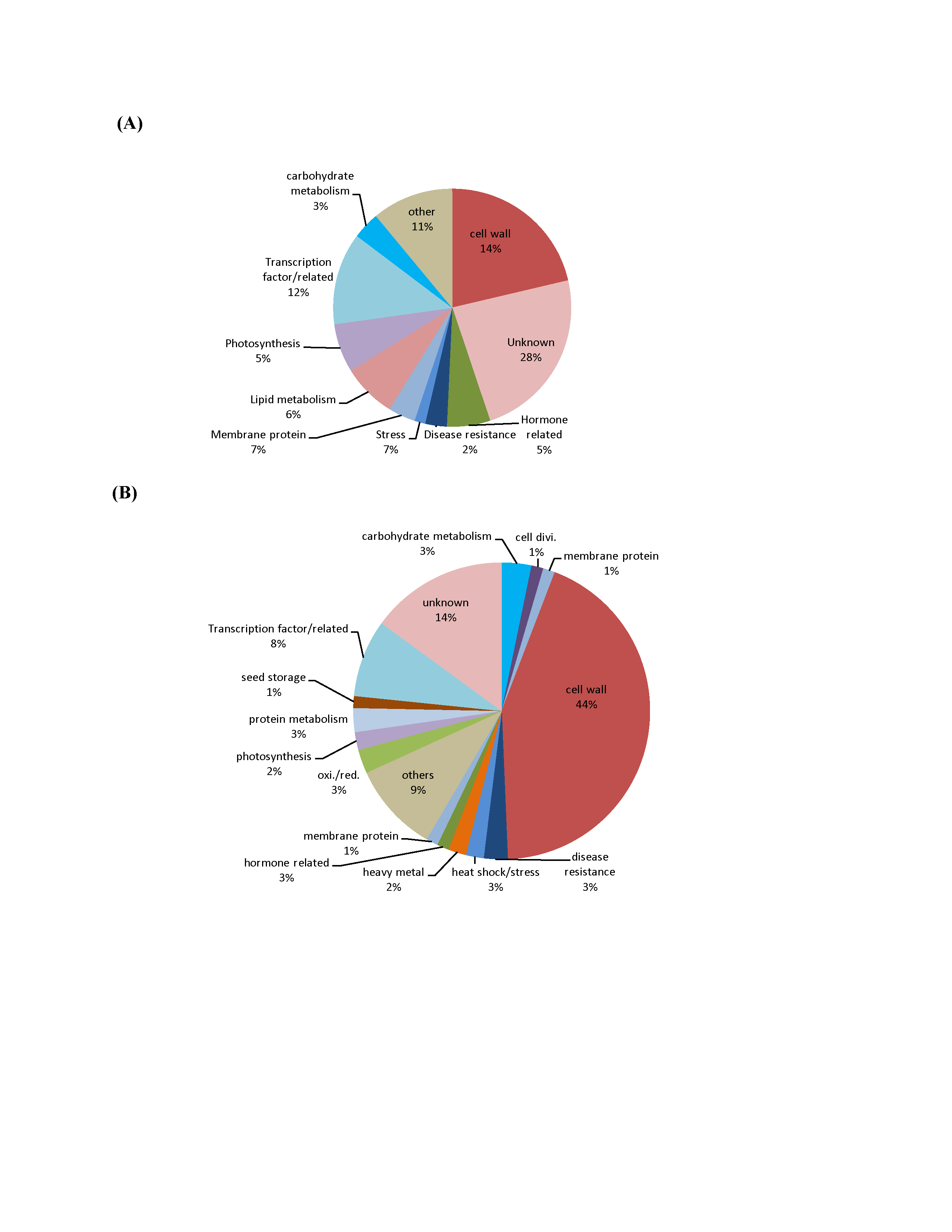

Supplement: Figure S12 — The Distribution of Differentially Expressed Genes in Seed Coats of Either (A) Clark or (B) Harosoy Backgrounds at the 100–200 mg Seed Weight Stage. The number of differentially expressed genes (≥5RPKM, ≥2 fold differential expression, p-value ≤0.05) was (A) 173 genes in Clark isolines and (B) 156 genes in Harosoy isolines at 100–200 mg seed weight stage. In both backgrounds, one of the major categories was related to the cell wall. (TIFF) [file pone.0096342.s012.tiff]

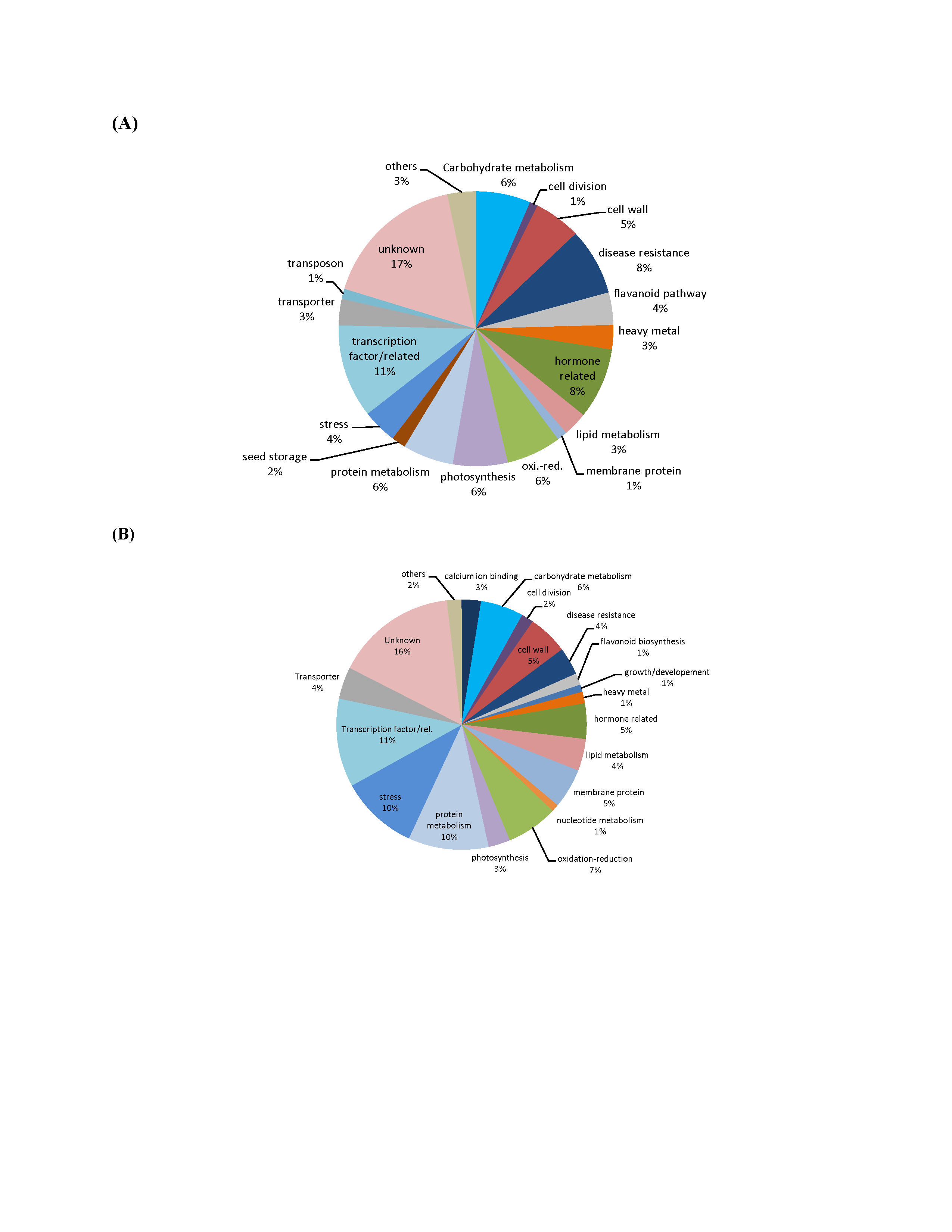

Supplement: Figure S13 — The Distribution of Differentially Expressed Genes in Seed Coats of (A) Clark or (B) Harosoy Backgrounds at the 400–500 mg Seed Weight Stage. The number of differentially expressed genes (≥5RPKM, ≥2 fold differential expression, p-value ≤0.05) was (A) 417 genes in Clark isolines and (B) 1068 genes in Harosoy isolines. In both backgrounds, the cell wall related genes occupied 5% of the chart area. (TIFF) [file pone.0096342.s013.tiff]

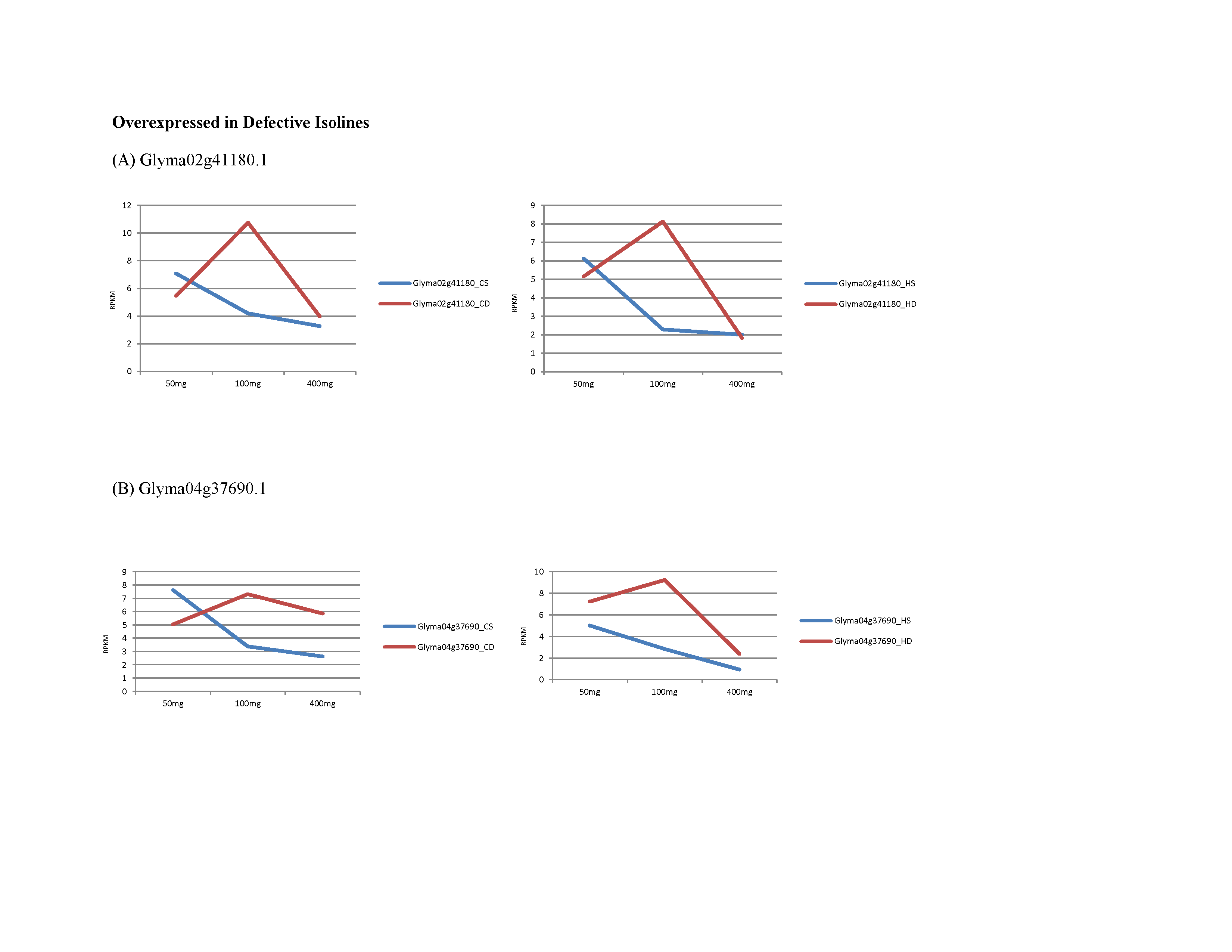

Supplement: Figure S14 — The Expression Pattern of Differentially Expressed Transcription Factor Genes That Showed Higher Expression in the Seed Coat of Defective Isolines in Both Clark and Harosoy Background. Overexpressed in defective isolines. CS: Clark Standard, CD: Clark Defective, HS: Harosoy Standard, HD: Harosoy Defective. (TIF) [file pone.0096342.s014.tif]

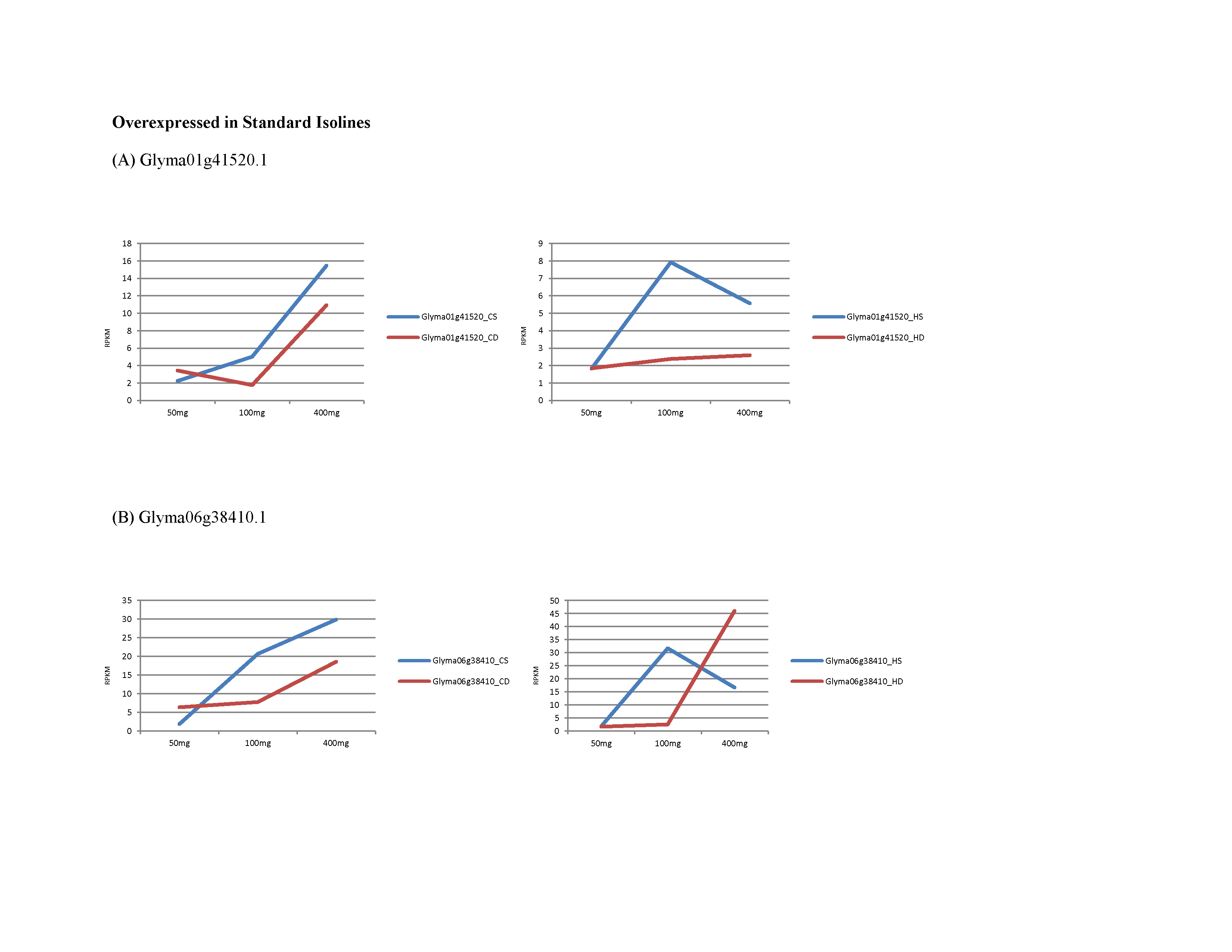

Supplement: Figure S15 — The Expression Pattern of Differentially Expressed Transcription Factor Genes That Showed Higher Expression in the Seed Coat of Standard Isolines in Both Clark and Harosoy Background. Overexpressed in standard isolines. CS: Clark Standard, CD: Clark Defective, HS: Harosoy Standard, HD: Harosoy Defective. (TIF) [file pone.0096342.s015.tif]

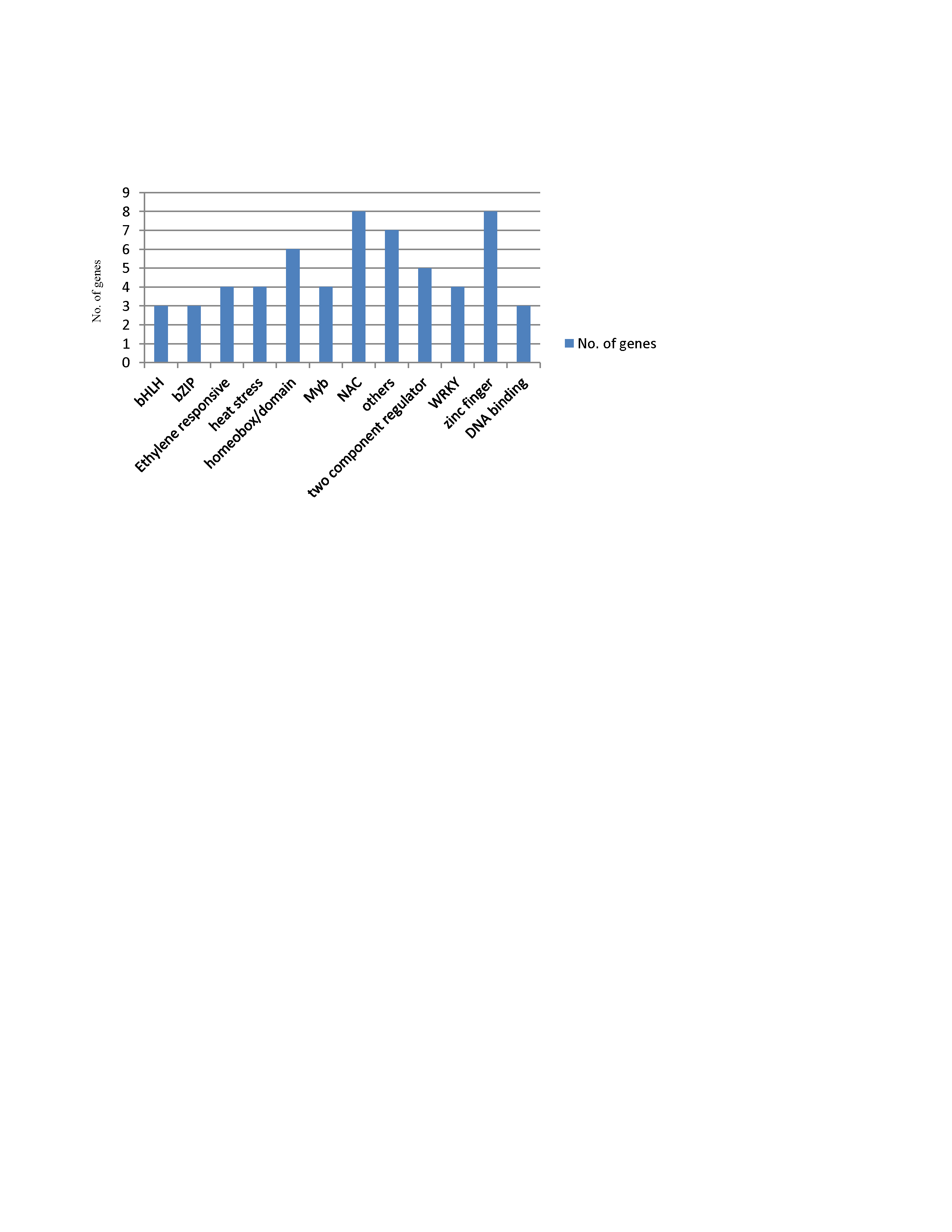

Supplement: Figure S16 — The Distribution of Differentially Expressed Transcription Factor Genes into Different Classes in Clark at the 50–100 mg Seed Weight Stage. The 59 differentially expressed transcription factor genes (≥5RPKM, ≥2 fold differential expression, p-value ≤0.05) were divided into 12 classes based on functional annotations. (TIFF) [file pone.0096342.s016.tiff]

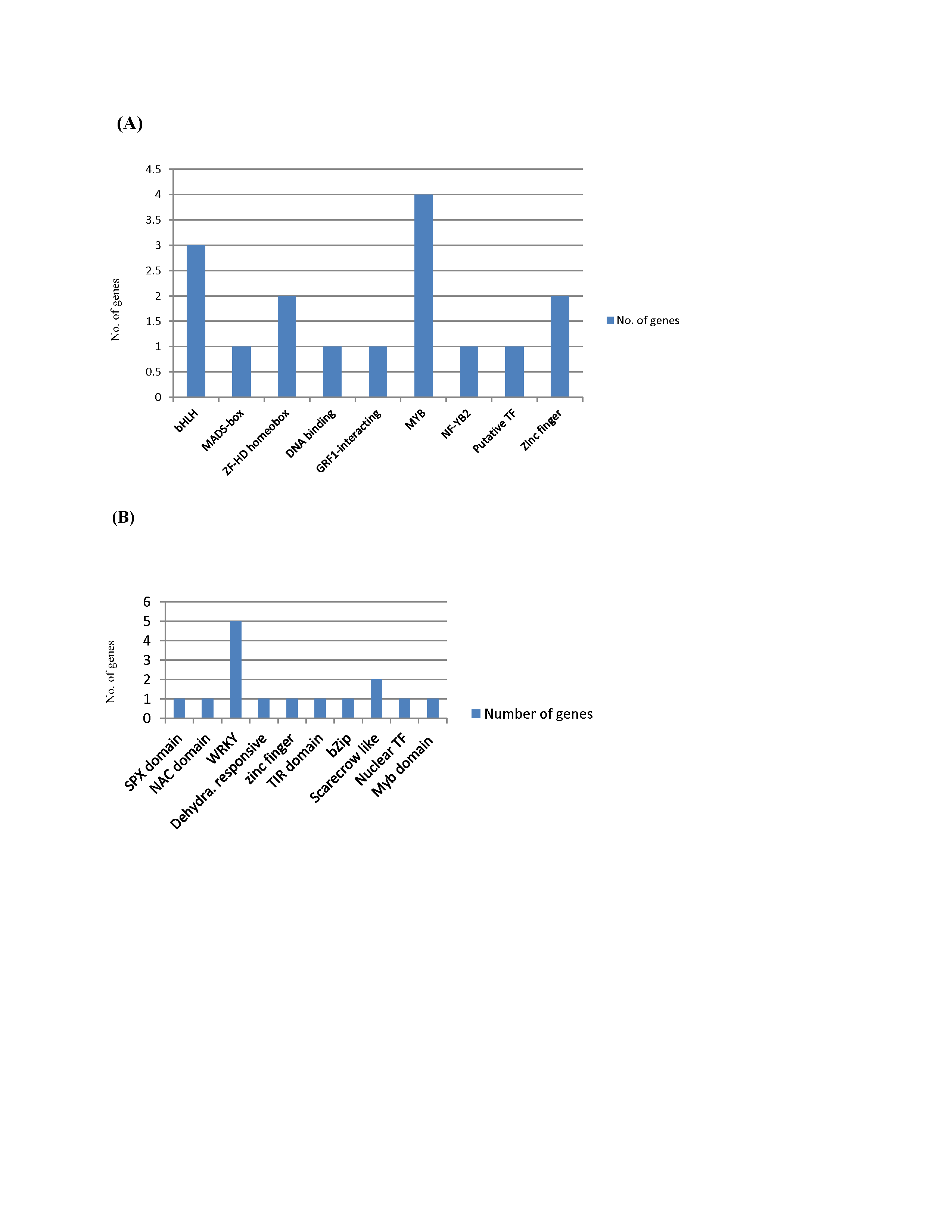

Supplement: Figure S17 — The Distribution of Differentially Expressed Transcription Factor Genes in (A) Clark or (B) Harosoy Backgrounds at the 100–200 mg Seed Weight Stage. (A) The 16 differentially expressed transcription factor genes (≥5RPKM, ≥2 fold differential expression, p-value≤.05) were divided into 9 different classes (B) The 12 differentially expressed transcription factor genes (≥5RPKM, ≥2 fold differential expression, p-value ≤0.05) were divided into 10 different classes based on functional annotation. (TIFF) [file pone.0096342.s017.tiff]

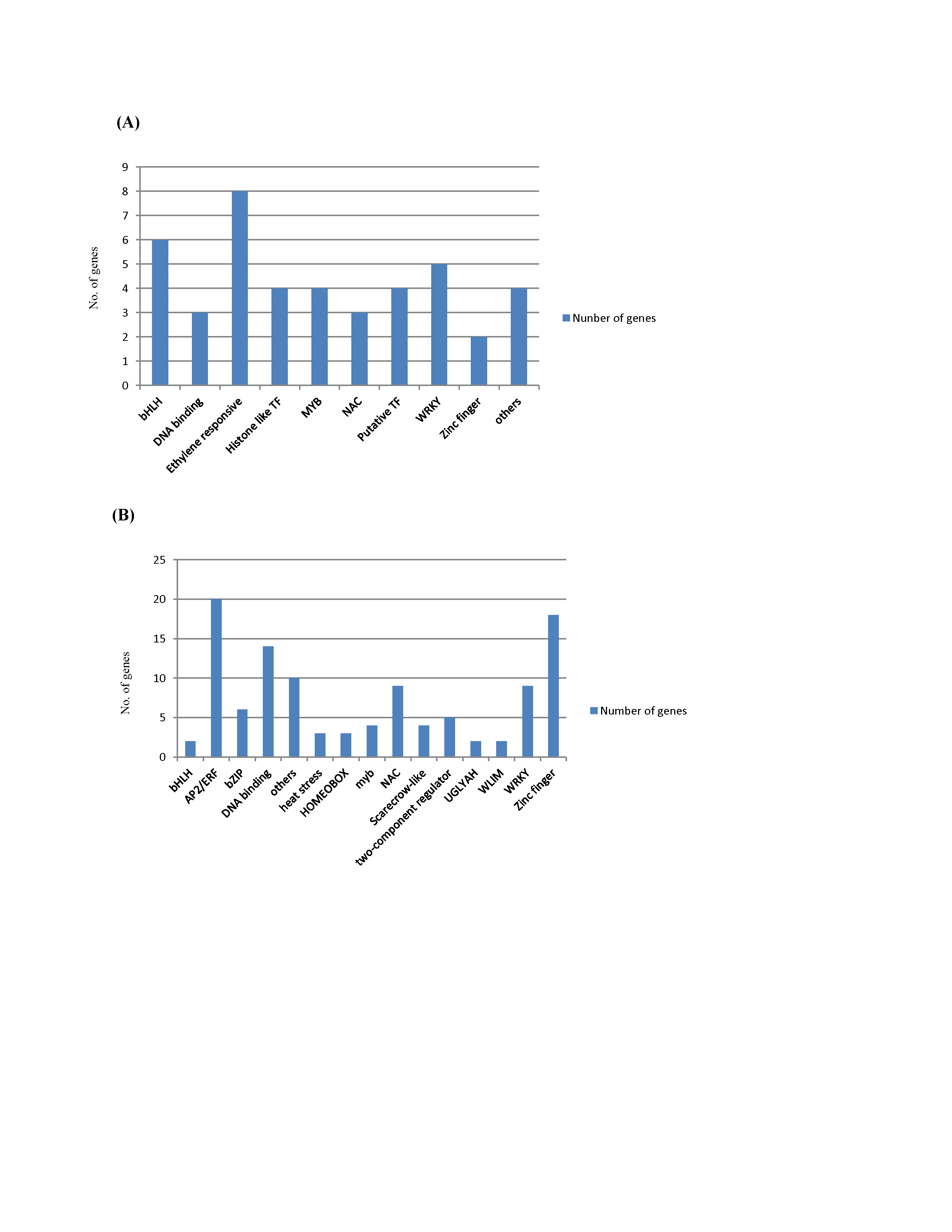

Supplement: Figure S18 — The Distribution of Differentially Expressed Transcription Factor Genes in Different Classes in (A) Clark and (B) Harosoy Backgrounds at the 400–500 mg Seed Weight Stage. (A) The 43 differentially expressed transcription factor genes (≥5RPKM, ≥2 fold differential expression, p-value ≤.05) were divided into 10 different classes (B) The 111 differentially expressed transcription factor genes (≥5RPKM, ≥2 fold differential expression, p-value ≤0.05) were divided into 15 different classes based on functional annotation. (TIFF) [file pone.0096342.s018.tiff]
