# Supplementary material for: RNA-Seq Profiling of a Defective Seed Coat Mutation in Glycine max Reveals Differential Expression of Proline-Rich and Other Cell Wall Protein Transcripts
Source: PLoS One. 2014 May 14;9(5):e96342. doi: 10.1371/journal.pone.0096342 (PMC4020777; doi:10.1371/journal.pone.0096342)
Supplement: Table S1 — The Total Number of Expressed Genes in Seed Coats of the Different Isolines. The total number of expressed genes at ≥10 RPKM or ≥1 RPKM in different backgrounds at different seed weight stages are presented in this table. CS: Clark standard, CD: Clark defective, HS: Harosoy Standard and HD: Harosoy defective. (DOCX) [file pone.0096342.s019.docx]

| **Genotype**  **(Seed Weight)** | **Number of Expressed Genes** | |
| --- | --- | --- |
|  | ≥10 RPKM | ≥1RPKM |
| CS (50 mg) | 13241 | 40125 |
| CD (50mg) | 13926 | 40803 |
| CS (100mg) | 13349 | 40877 |
| CD (100mg) | 13427 | 40966 |
| CS (400mg) | 14182 | 38486 |
| CD (400mg) | 13914 | 38070 |
| HS (50mg) | 13551 | 40437 |
| HD (50mg) | 13317 | 40345 |
| HS (100 mg) | 14036 | 41347 |
| HD (100mg) | 13308 | 41891 |
| HS (400mg) | 13569 | 37685 |
| HD (400mg) | 13165 | 37221 |

**Table S1 The Total Number of Expressed Genes in Seed Coats of the Different Isolines.** The total number of expressed genes at ≥10 RPKM or ≥1 RPKM in different backgrounds at different seed weight stages are presented in this table. CS: Clark standard, CD: Clark defective, HS: Harosoy Standard and HD: Harosoy defective
